# The gut microbiota composition of *Trichoplusia ni* is altered by diet and may influence its polyphagous behavior

**DOI:** 10.1038/s41598-021-85057-0

**Published:** 2021-03-11

**Authors:** M. Leite-Mondin, M. J. DiLegge, D. K. Manter, T. L. Weir, M. C. Silva-Filho, J. M. Vivanco

**Affiliations:** 1 Departmento de Genética, Escola Superior de Agricultura Luiz de Queiroz, Universidade de São Paulo, Av. Pádua Dias, 11, Piracicaba, SP 13418-900 Brazil; 2 USDA, Center for Agricultural Research Services, Soil-Plant-Nutrient Research Unit, Fort Collins, CO USA; 3 Department of Food Science and Human Nutrition, Colorado State University, Fort Collins, CO USA; 4 Department of Horticulture and Landscape Architecture, Colorado State University, Fort Collins, CO USA

**Keywords:** Applied microbiology, Microbial communities, Microbiome, Entomology

## Abstract

Insects are known plant pests, and some of them such as *Trichoplusia ni* feed on a variety of crops. In this study, *Trichoplusia ni* was fed distinct diets of leaves of *Arabidopsis thaliana* or *Solanum lycopersicum* as well as an artificial diet. After four generations, the microbial composition of the insect gut was evaluated to determine if the diet influenced the structure and function of the microbial communities. The population fed with *A. thaliana* had higher proportions of *Shinella*, *Terribacillus* and *Propionibacterium,* and these genera are known to have tolerance to glucosinolate activity, which is produced by *A. thaliana* to deter insects. The population fed with *S. lycopersicum* expressed increased relative abundances of the *Agrobacterium* and *Rhizobium* genera. These microbial members can degrade alkaloids, which are produced by *S. lycopersicum*. All five of these genera were also present in the respective leaves of either *A. thaliana* or *S. lycopersicum*, suggesting that these microbes are acquired by the insects from the diet itself. This study describes a potential mechanism used by generalist insects to become habituated to their available diet based on acquisition of phytochemical degrading gut bacteria.

## Introduction

*Trichoplusia ni* (Hübner, 1803) (Lepidoptera; Noctuidae) larvae are generalist herbivores and considered to be critical agricultural pests, which feed on more than 100 species of plants including tomato, potato, corn, cotton, and many other crops^1^. Furthermore, *T. ni* is one of the most important field and greenhouse pests in North America and has been reported to cause economic damage across a widespread geographic range from Canada to Mexico^2^. It is known that herbivorous insects have genetic adaptations that allow for the expression of digestive enzymes, which metabolize different plant compounds that could become toxic to the insect^3,4^. In addition, a large proportion of gut bacteria are presumably responsible for an insect’s ability to resist or detoxify phytotoxins^1^.

The investigation of the gut microbiota within insect larvae has increased substantially in recent years^5–10^. A combination of high throughput sequencing with phylogenetic analyses has revealed a high diversity of bacterial communities living in the intestine of a variety of insects^8–10^. Unlike mammalian guts, insect microbiota profiles are usually dominated by Proteobacteria followed by Firmicutes^8–10^. Gamma-proteobacteria are able to increase insect tolerance to high temperatures and mediate specificity toward certain host plants^11^. Proteobacteria living in the gut of Hymenopteran insects have been shown to improve host resistance to pathogenic parasites^12^. The bumble bee susceptibility to the parasite *Crithidia bombi* was influenced by the composition of the gut microbiota. This result substantiates the role of the microbiota of social bees in gut homeostasis and resistance against parasites and diseases^13^. Some Proteobacteria, such as *Wolbachia* sp*.,* can induce male-killing, feminization, and parthenogenesis in insect models such as *Asobara japonica* (a larval parasitoid of drosophila flies) (Förster, 1862) and *Armadillidium vulgare* (pill woodlouse) (Latreille, 1804)^14,15^. Furthermore, both direct and trans-generational effects were observed to be caused by the gut microbiota of *Drosophila melanogaster* (Meigen, 1830)^16^.

It has been found that an insect’s diet, environmental habitat, and developmental stage modified the insect host’s-associated gut microbiota^8,10,17,18^. For instance, a diet of beans (containing cyanogenic glycosides) increased the mortality of *Spodoptera littoralis* (Boisduval, 1833) larvae, but when the larvae later ingested a diet rich in barley; their gut communities were recovered^19^. This study showed that the initial microbiota of the bean-fed *S. littoralis* larvae was composed almost entirely by two bacteria, *Enterococcus mundii* (25%) and *Pantoea agglomerans* (50%). After the larvae were fed with barley, the detected gut microbiota presented *Clostridia* and *Enterococcus casseliflavus,* exemplifying the influence of the host’s diet on colonization of dominant taxa in their intestinal tract^19^. Termite studies have demonstrated a correlation between resident bacterial communities and effective lignocellulosic degradation of administered plant diets^20^. As such, the components present in the plant diet were presumed to modulate host microbial communities^20^. In addition to taxonomic structure, other studies have shown that changes in the termite diet results in altered functional genomics of the bacteria present within the termite’s gut^21^.

Insect gut microbiota have been the subject of experiments seeking to understand the relationship between herbivore resistance to phytochemical compounds within their diets as well as toward pathogenic microbes. To exemplify this, *Bacillus thuringiensis* has shown variations in its pathogenicity against insects, depending on the intestinal microbiota associated with its host^22^. The same type of protective microbiome effect can be seen against *Baculoviral* infections. The insect's natural microbiota can play a protective role against *Baculoviral* infections via stimulation of the basal level of insect immunity in larvae of *Spodoptera exigua* (Hübner, 1808)^23^. Furthermore, some endogenous intestinal bacteria in insects can detoxify plant secondary metabolites that may be harmful to the host^11,17^. The gut microbiota of insects is also involved in the insect's nutrition, maintenance of fitness^17,22^, homeostasis against plant defense systems^24^ and may be targeted in novel strategies to control insect pests. Additionally, different populations of *Plutella xylostella* (Linnaeus, 1758) fed with radish and peas showed distinct metabolic profiles in their guts, which correlated with intestinal bacteria^25^.

In this study, we sought to understand how different specific diets may change the gut microbiota of *T. ni* and whether these microbial changes might contribute to the generalist feeding behavior of the insect. To accomplish this, we fed *T. ni* with three distinct diets for several generations and then explored the composition of their gut microbiota as well as the microbial profiles associated with the various food sources.

## Results

### Food preference

**Figure 1:**
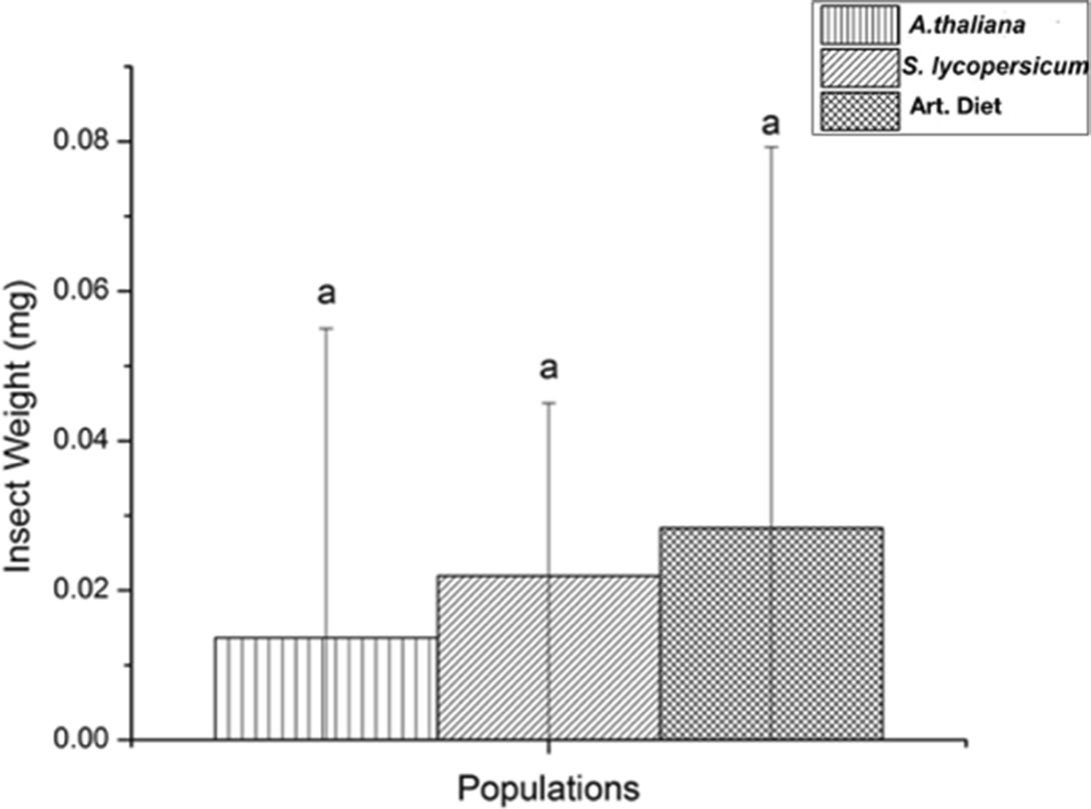
The difference in the mean insect weight of *Trichoplusia ni* after 24 h of preferential feeding with the possibility of choice. The different populations are represented in the x-axis. ANOVA (F test). Each letter at the top of the bars represents significant mean differences by the Tukey test at the p < 0.05 significance level.

**Figure 2:**
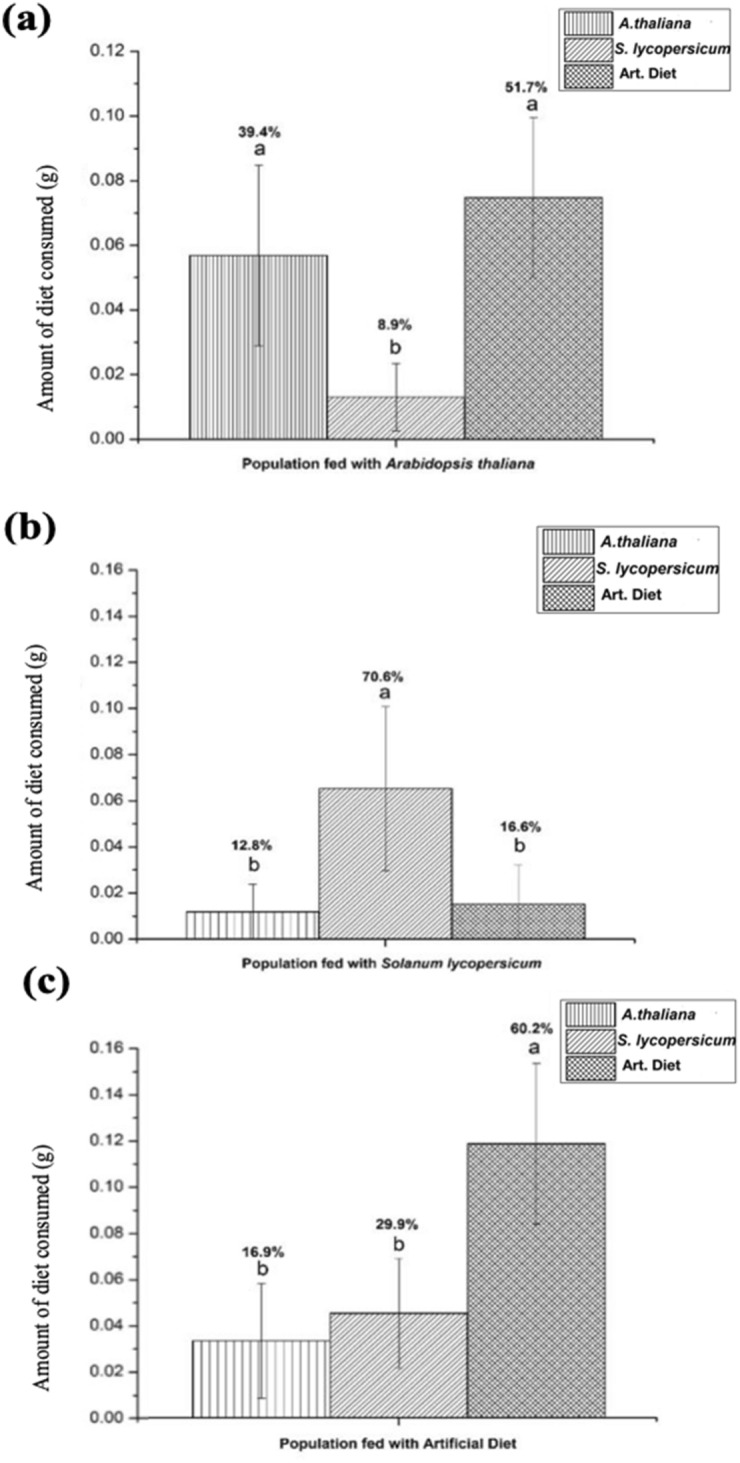
Weight of each diet administered after exposure to insect populations during the feeding preference experiment. Insect populations used in this assay were previously fed with a specific diet of *A. thaliana* leaves (**a**), *S. lycopersicum* leaves (**b**) or with an artificial diet (**c**). Each bar corresponds to the average weight change of each diet after feeding (n = 10). The different populations are represented on the x-axis. ANOVA (F test) were conducted to determine significance. Each letter at the top of error bars represents the mean difference after a *Tukey post-hoc* test at the p < 0.05 significance level. The percentages above the bars in each graph correspond to percent-consumed of each food, per colony during the experiment.

The food preference study was carried out for three different populations of insects fed with differing diets (*Arabidopsis thaliana*, *Solanum lycopersicum*, or the artificial diet) after the three previous progenitor generations were fed with only one of each forementioned diet. At the end of the food preference test, no significant differences were found in mean larval weight among the three insect groups (Fig. 1). Furthermore, populations of *T. ni* fed with *A. thaliana* leaves showed a preference towards *A. thaliana* leaves and the artificial diet (Fig. 2a) over *S. lycopersicum*. The insects fed with leaves of *S. lycopersicum* preferred the *S. lycopersicum* diet over both *A. thaliana* and the artificial diet (Fig. 2b). Lastly, the insects fed the artificial diet showed a preference of the artificial diet over both *A. thaliana* and *S. lycopersicum* leaves (Fig. 2c).

### Illumina MiSeq data

After filtering reads and removing amplicon sequence variants (ASVs) with singleton representation in the data set*,* Illumina MiSeq sequencing generated a total of 1,854,660 sequencing reads resulting in an average of 102,592 reads per sample, and each sample was comprised of pooled insect guts (n = 20). The Good's coverage of each sample, which reflects the captured diversity (Good’s coverage = number of singleton ASVs/total abundance sums of all ASVs) was higher than 98.01% for all samples. Therefore, the sequencing depth was adequate to assess the diversity of bacterial communities within the *T. ni* insect gut, *A. thaliana*, *S. lycopersicum* leaves, as well as within the artificial diet.

### Resident microbiome composition of each diet

**Figure 3:**
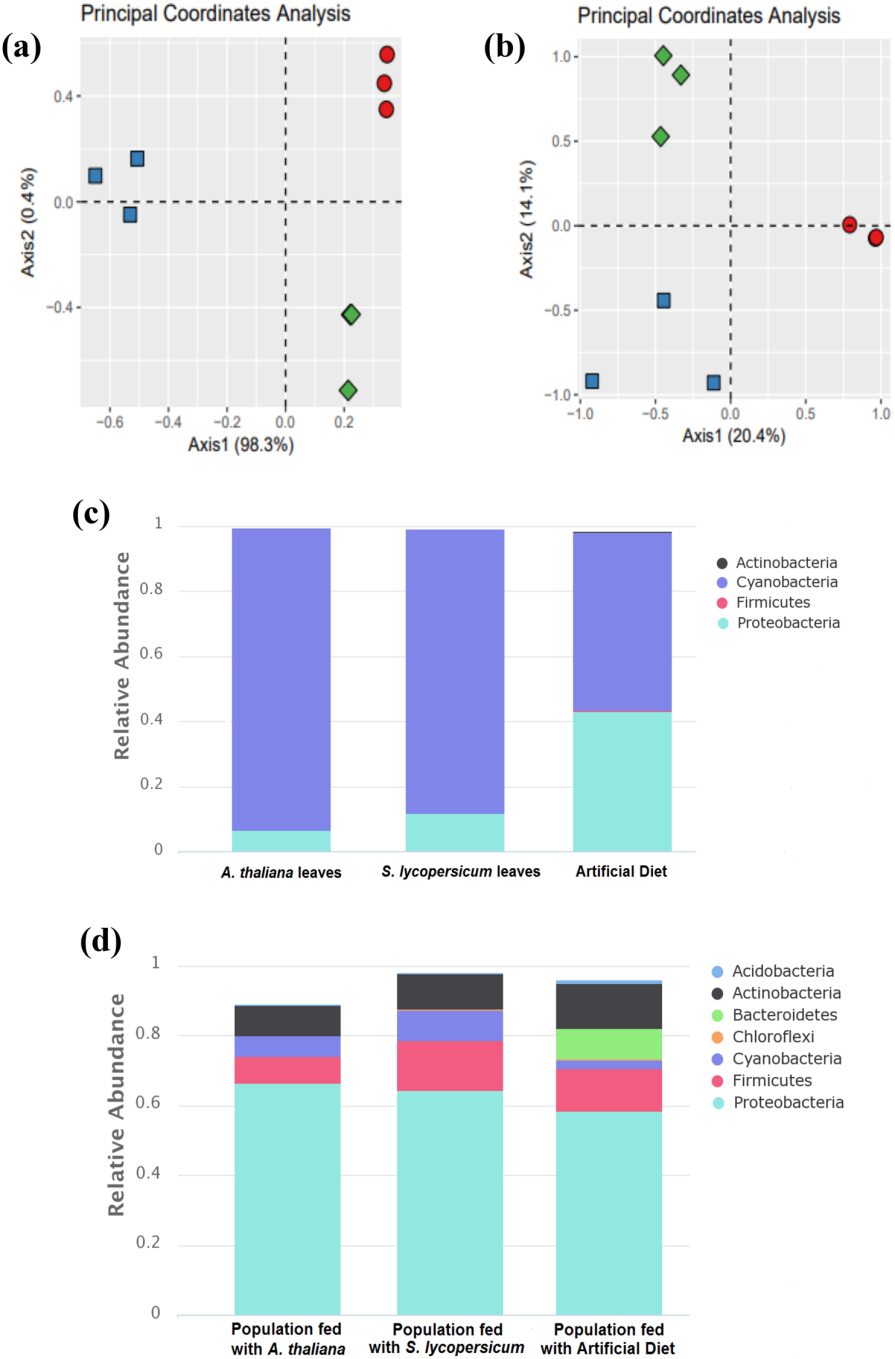
(**a**) Principal coordinate analysis (PCoA) of pairwise dissimilarities (Bray–Curtis index) for bacterial communities in *A. thaliana* leaves (red circles), *S. lycopersicum* leaves (green rhombi) and in the artificial diet (blue squares). (**b**). Principal coordinate analysis (PCoA) of pairwise dissimilarities (Bray–Curtis index) for bacterial communities in guts of *Trichoplusia ni* derived from populations fed with each different diet. (**c**). Bacterial relative abundance measures from ASVs classified at the phyla level present in each diet (*A. thaliana* leaves*, S. lycopersicum* leaves or artificial diet*)*. ANCOVA (p < 0.05). (**d**). Bacterial relative abundances of ASVs classified at the phyla level in *Trichoplusia ni* guts samples from populations of *Trichoplusia ni* fed with specific diets. The size of each segment in the (**c**) and the (**d**) chart is proportional to the relative abundance of the ASVs assigned to each phylum.

The bacterial composition was found to be distinct among the three diets based on perMANOVA (999 permutations) and principal coordinates of analysis (PCoA). Samples from *A. thaliana* leaves, *S. lycopersicum* leaves and the artificial diet formed distinct and significantly different clusters (p = 0.004) (Fig. 3a).

According to ANcOVA analysis, some bacterial taxa were shared at the phyla level among the different diets such as Bacteroidetes, Cyanobacteria, Proteobacteria, and Firmicutes (Fig. 3c). For all diets, Cyanobacteria (93–55% relative abundance) and Proteobacteria (7–43% relative abundance) were the predominant phyla (Table 1). Additionally, a small percentage of Chloroflexi (0.02%), Gemmatimonadetes (0.01%), Firmicutes (0.3%) and Planctomycetes (0.01%) were present in the artificial diet. The relative abundance values of detected taxa, classified to the phyla level, were significantly different (p < 0.05) between diet-associated bacterial communities.

**Table 1:** Bacterial relative abundances from plant and artificial diets used to feed *Trichoplusia ni* populations.

Phylum	A. thaliana leaves (%)	S. lycopersicum leaves (%)	Artificial Diet (%)	p-value
Cyanobacteria*	93	87	55	< 0.0001
Proteobacteria*	7	12	43	< 0.0001
Firmicutes*	0	0	0.3	0.0005
Actinobacteria*	0	0	0.1	0.0009
Unclassifiedclassifed*	0	1	1.6	0.0002

ANCOVA univariate test (P < 0.05).

Bacterial relative abundance percentages were determined by comparing the numbers of ASVs classified at the phyla level to all other detected microbiota in each community. Asterisks indicate statistically significant differences between abundance values detected from all populations to p < 0.05.

We conducted an additional ANcOVA to determine differences in bacterial populations able to be classified at the genus level. Interestingly, there were significant differences (p < 0.05) in relative abundances in each of the diet-communities analyzed. The following genera were observed to be significantly different at the genus level, across all diets: *Agrobacterium* (p < 0.001), *Azospirillum* (p = 0.023), *Delfitia* (p < 0.001), *Propionibacterium* (p = 0.027), *Pseudomonas* (p < 0.001), *Sphingobium* (p = 0.006), and *Streptomyces* (p = 0.0116) (Supplementary Table S1).

### Gut microbiome composition of *T. ni* populations fed with different diets

The bacterial composition present within the insect gut of *T. ni* fed with specific diets presented significant differences among microbial communities (perMANOVA; p = 0.041). The comparison was based on Bray–Curtis dissimilarity index and visualized by PCoA (Fig. 3b). An ANcOVA was used to determine the most abundant groups present in *T. ni* regardless of diet administered: Proteobacteria, Actinobacteria, Firmicutes, Cyanobacteria, Acidobacteria, Bacteroides and Chloroflexi (Fig. 3d). In Table 2 we show the percent abundances for each detected phylum present within the gut of *T. ni* across the different populations analyzed. However, no significant phyla-level differences were detected after an ANcOVA test of gut samples from different populations of *T. ni* (Table 2).

**Table 2:** Relative abundances of bacteria detected within gut samples of different *Trichoplusia ni* populations.

Phylum	Population fed with A. thaliana leaves (%)	Population fed with S. lycopersicum leaves (%)	Population fed with Artificial Diet (%)	p-value
Proteobacteria	66.22	64.25	58.27	0.883
Actinobacteria	8.56	9.91	13.07	0.39
Firmicutes	7.73	14	12.06	0.124
Cyanobacteria	5.63	8.94	2.52	0.58
Acidobacteria	0.37	0.3	0.81	0.608
Bacteroidetes	0	0	8.68	0.402
Chloroflexi	0	0.17	0.15	0.467
Unclassified	11.49	2.43	4.44	0.547

Bacterial relative abundance percentages were determined by comparing the numbers of ASVs classified at the phyla level to all other detected microbiota within each community. For all phyla, no statistically significant differences were detected between the different communities sampled (p > 0.05).

**Figure 4:**
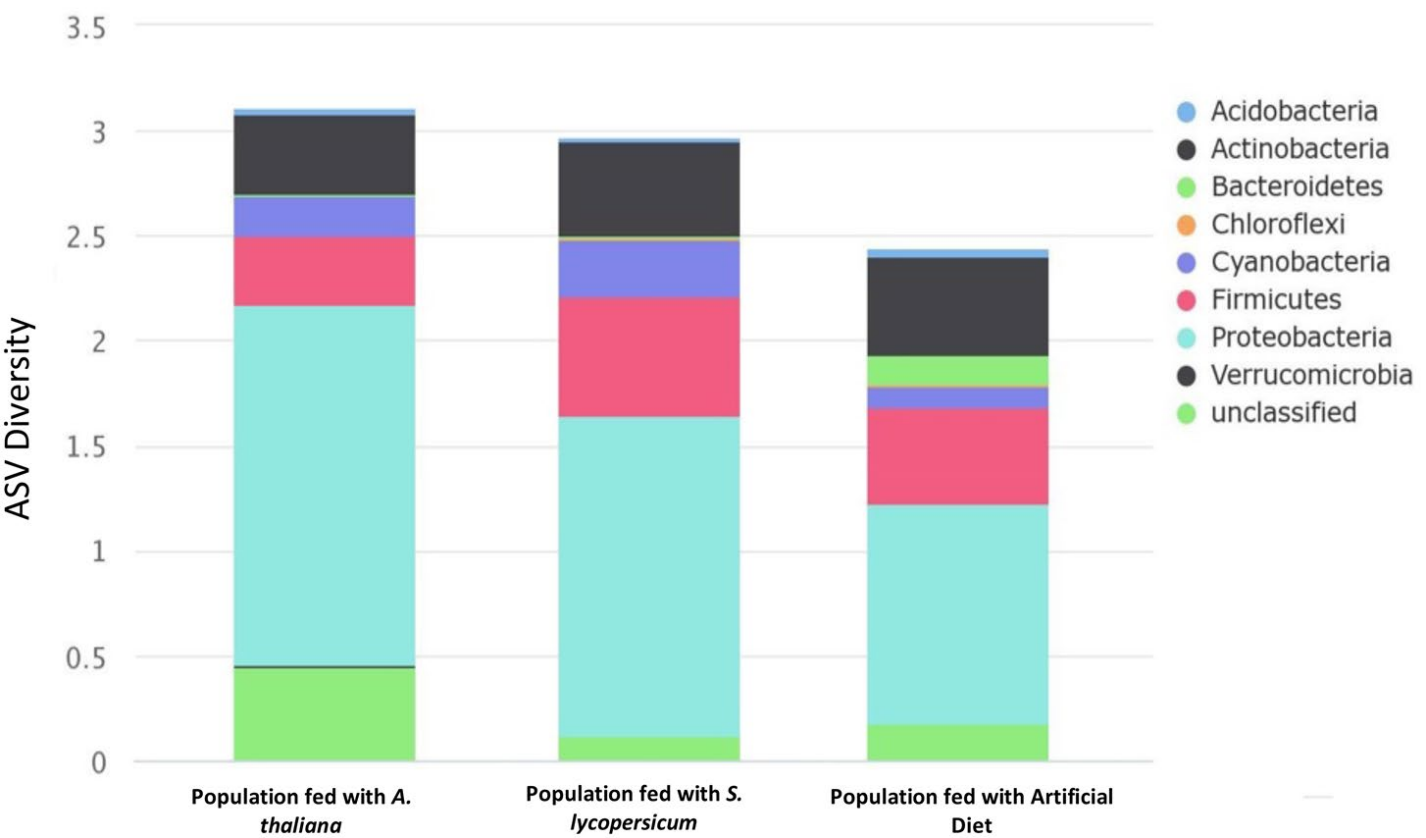
Bacterial phylogenetic diversity of microbiota detected in the guts of *Trichoplusia ni* populations fed with exclusive diets. Bacterial diversity from each population corresponds the numbers of ASVs classified at the phyla level.

Another ANcOVA analysis at the genus and family levels revealed significant differences within gut microbiota communities of *T. ni* fed with the different diets. Abundances of the genera *Achromobacter* (p = 0.039), *Enterococcus* (p = 0.042), *Mesorhizobium* (p < 0.001), *Microbacterium* (p = 0.018), *Shinella* (p < 0.001), *Veillonella* (p = 0.037), along with families *Comamonadaceae* (p = 0.006), *Streptomycetaceae* (p = 0.03), *Alcaligenaceae* (p < 0.001) and *Planococcaceae* (p = 0.021) presented significant differences between insect populations fed with each distinct diet. The relative abundance of bacteria detected within the gut of *T. ni* in each treatment (classified to the genus level) can be visualized in Supplementary Table S2. Shannon’s phylogenetic diversity shows that the *T. ni* populations fed with *A. thaliana* leaves resulted in the highest species diversity, followed by the population fed only with *S. lycopersicum* leaves and lastly from the artificial diet (Table 3). Phylum level bacteria diversity can be visualized in Fig. 4.

**Table 3:** Phyla-level bacterial diversity in each diet administered *Trichoplusia ni* during experimentation.

Phylum	Population fed with A. thaliana leaves (%)	Population fed with S. lycopersicum leaves (%)	Population fed with Artificial Diet (%)	p-value
Acidobacteria	0.0710	0.056	0.104	0.748
Actinobacteria	1.147	1.337	1.429	0.698
Bacteroidetes	0.016	0.053	0.412	0.348
Chloroflexi	0	0.033	0.024	0.427
Cyanobacteria	0.583	0.787	0.286	0.552
Firmicutes*	0.976	1.723	1.393	0.039
Proteobacteria	5.165	4.568	3.139	0.163
unclassified	1.354	0.358	0.526	0.549
Total	9.312	8.915	7.313	0.945

*ANCOVA univariate test (P < 0.05).

The amount of bacterial diversity observed in each population corresponds to the numbers of ASVs classified at the phyla level to visualize sample alpha diversity with *Shannon’s* diversity index in the row described “total”. Asterisks indicate statistically significant differences among all pairs of values. (*) *p* < *0.05.*

**Figure 5:**
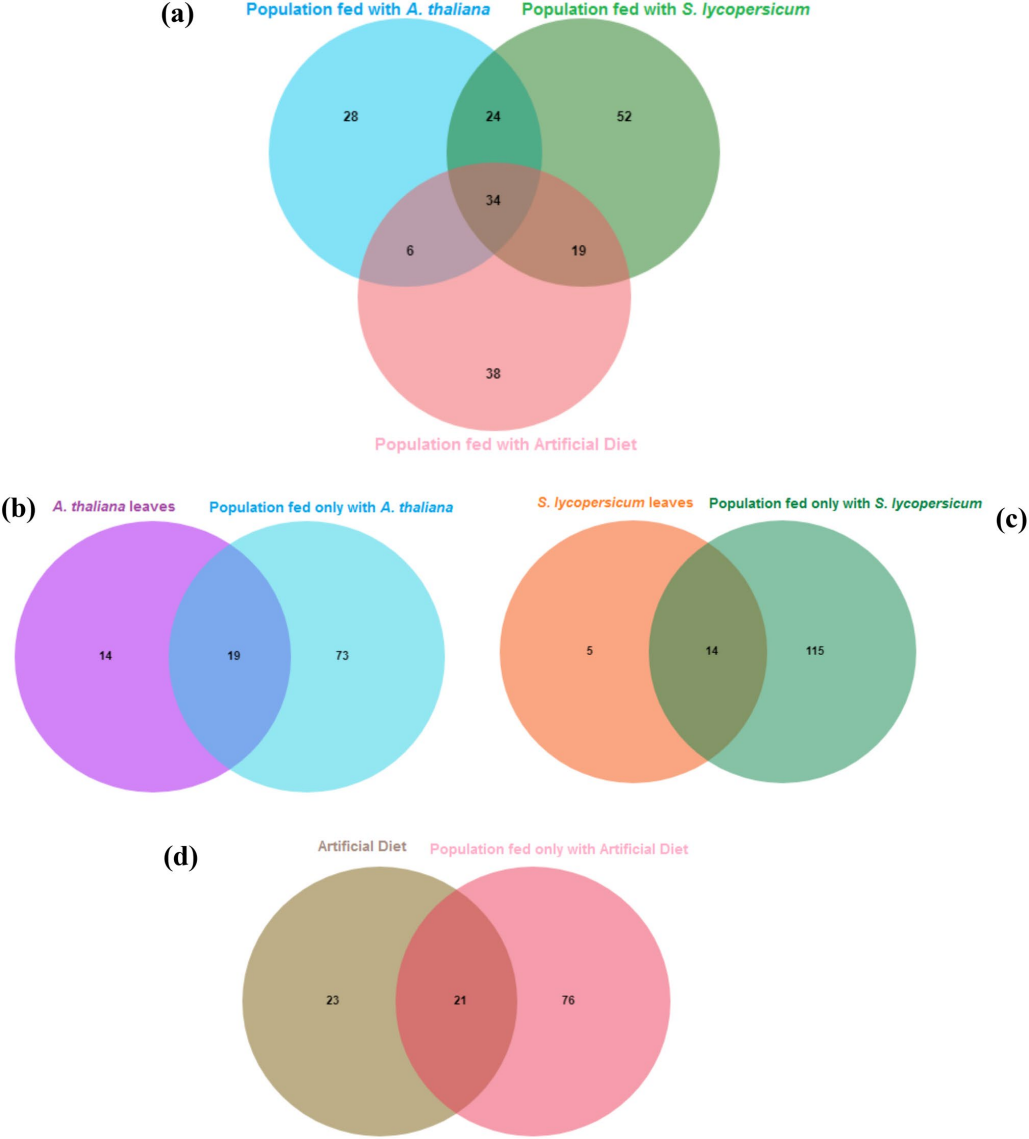
Venn diagrams showing distribution of shared and unique ASVs assigned at the genus level among the different *T.ni* populations fed with exclusive diets over four cycles. (**a**) Venn diagram showing common or unique ASVs among insects fed with *A. thaliana* leaves, *S. lycopersicum* leaves, or the artificial diet. (**b**) Common or unique ASVs detected in *A. thaliana* leaves and guts of the insect population fed with *A. thaliana* leaves. (**c**) Common or unique ASVs detected in *S. lycopersicum* leaves and guts of the insect population fed with *S. lycopersicum* leaves. (**d**) Common or unique ASVs detected in artificial diet and guts of the insect population fed with the artificial diet. leaves. The names data about the common or unique genera in all Venn diagrams are presented in Supplementary Table S3.

At the genus level, the *A. thaliana* fed populations were associated with 28 unique genera. We found 52 unique genera for the population fed with leaves of *S. lycopersicum,* and the population fed only with artificial diet showed 38 unique taxa at the genus level (Fig. 5a) using ANcOVA analyses. The following bacterial genera were detected at significantly different relative abundance values within gut microbiome samples of *T. ni* fed with different diets: *Achromobacter* (p = 0.004), *Bacillus* (p = 0.006), *Enterococcus* (p = 0.011), *Gemella* (p = 0.027), *Mesorrhizobium* (p = 0.032), *Propionibacterium* (p = 0.0427), *Streptococcus* (p = 0.049), and *Veillonella* (p < 0.001) (Supplementary Table S3). Furthermore, 34 taxa classified to the genus level were shared among all three insect populations. Additionally, 24 genera were shared between the *A. thaliana* and *S. lycopersicum* populations, 6 between *A. thaliana* and artificial diet populations, and 19 among *S. lycopersicum* and artificial diet population (Fig. 5a and Supplementary Table S3). The complete list of reads that are shared and those that are unique to each individual population can be visualized in Supplementary Table S3).

### Overlap of diet source and T. ni bacterial communities

After ANcOVA analyses it was found that the bacteria detected in the gut of *T. ni* populations fed with *A. thaliana* leaves shared 19 common genera with resident communities detected on leaves of *A. thaliana* (Fig. 5b). Of these 19 genera, ten were exclusively present within samples of *A. thaliana*, whether sampling was from the insect gut or the leaf tissue of *A. thaliana*. Taxa uniquely present within *A. thaliana* communities included the families Bradyrhizobiaceae, Hyphomicrobiaceae, Oxalobacteraceae, Rhizobiaceae, Rhodospirillaceae along with the genera *Rhodoplanes*, *Mesorhizobium*, *Shinella*, and *Streptomyces* (Supplementary Table S3).

For the populations fed with *S. lycopersicum* leaves, 14 genera observed to be present in the insects’ gut were also common to samples from leaves of *S. lycopersicum* (Fig. 5c). Of these 14 genera, seven were specific to samples from *S. lycopersicum* leaves and found in insect guts or leaf tissue. The taxa uniquely present in *S. lycopersicum* samples included the families Caulobacteraceae, Comamonadaceae, along with the genera *Chryseobacterium, Delftia, Rhizobium, Sphingomonas,* and *Agrobacterium* (Supplementary Table S3).

The population fed only with the artificial diet resulted in 21 genus level bacteria shared between insect guts and physical samples of the artificial diet (Fig. 5d). Only, 12 genera were specific to samples analyzed from the artificial diet itself or insects fed with the diet. Taxa unique to the artificial diet samples included the families AKIW874, Gaiellaceae, Streptomycetaceae and the genera *Actinobaculum*, *Actinomadura*, *Aeromicrobium*, *Bacillus*, *Corynebacterium*, *Gemmatimonadetes*, *Geobacillus*, *Rhodanobacter*, and *Staphylococcus* (Supplementary Table S3).

The bacterial communities were significantly different based on the analysis of permANOVA (perMANOVA; p = 0.041). However, it is worth noting that according to the eigenvalues ​​of the PCoA axes (Fig. 3b), only a small percentage of the observed variation is explained by the diet.

### Effect of diet-source on gene-specific abundances from bacterial communities in the Trichoplusia ni gut

Our PICRUSt analysis was used to locate genes within the glycosidases category of the KEGG Enzymes database in the microbial community in order to predict the presence of enzymes that degrade glucosinolates or glycoalkaloids naturally produced within the administered diets (*A. thaliana* or *S. lycopersicum*) (Table 4, Supplementary Table S4)^24,26^.

**Table 4:** Effect of restrictive diets on the relative abundances of predicted gut bacterial gene function within *Trichoplusia ni* fed with different plant diets*.*

Genetic information		Relative abundance		p-value
Function	K-Orthology	Gut fed *A. thaliana*	Gut fed *S. lycopersicum*	
Alpha-amylase	EC.3.2.1.1	0.220	0.500	0.011
Alpha-mannosidase	EC.3.2.1.24	0.175	0.440	0.026
Beta-glucuronidase	EC.3.2.1.31	0.009	0.032	0.026
Alpha-L-rhamnosidase	EC.3.2.1.40	0.027	0.013	0.045
Pullulanase	EC.3.2.1.41	0.028	0.109	0.007
Alpha-n-acetylglucoasminidase	EC.3.2.1.50	0.001	0.005	0.002
Bifunctional autolysin	EC.3.5.1.28 3.2.1.96	0.003	0.009	0.012
Mannosyl-glycoprotein endo-beta-N-acetylglucosaminidase	EC.3.2.1.96	0.014	0.085	0.003

All displayed gene functions have been significantly increased or decreased as a result of restrictive diet administration to the *Trichoplusia ni* groups used in this study.

For the predicted functions of bacterial genes present in insects fed with *S. lycopersicum* leaves, the relative abundance values of glycosidase-enzymes were observed to be significantly higher when compared with *A. thaliana*-fed communities (Table 4). The predicted functional genes significantly increased in relative abundances when insects were fed with the *S. lycopersicum* diet were as follows: alpha-amylase (EC.3.2.1.1), alpha-mannosidase (EC.3.2.1.24), beta-glucuronidase (EC.3.2.1.31), pullulanase (EC.3.2.1.41) alpha-N-acetylglucoasminidase (EC.3.2.1.50), bifunctional sutolysin (EC.3.5.1.28 3.2.1.96) and mannosyl-glycoprotein endo-beta-N-acetylglucosaminidase (EC.3.2.1.96). For the predicted function of bacterial members of insects fed with *A. thaliana*, relative abundances of alpha-L-rhamnosidase (EC.3.2.1.40) was significantly increased.

### Weighted correlation network analysis (WGCNA) of co-occurring genera

We further analyzed the 16S amplicon sequence data in search of candidate genera expressing predicted functional gene mechanisms based on phylogeny to evade phytochemical toxicity of compounds present within each diet. A weighted correlation network analysis (Supplementary Fig. S2) revealed eight modules of co-occurring bacterial genera in the gut communities of insects for all treatments. All genera were assigned to a particular color-coded module. Genera from each gut community resulting in the highest kWithin values were indicative of the connectivity of each genus within its respective module, to all other co-occurring genera within the same module^27^. The analysis revealed two modules (green and red) that corresponded to correlation networks of gut communities from insects fed with leaves of *A. thaliana*; three modules (brown, blue, and pink) corresponding to correlation networks of gut bacterial communities from insects fed *Solanum lycopersicum* leaves; and another three modules (turquoise, red, and magenta) corresponding to correlation networks of gut bacterial communities of insects fed with the artificial diet. Of all modules, the green and brown modules expressed the highest value of eigengenes (0.607 and 0.309, respectively), and as such were further investigated for co-occurring genera present within insect guts fed *A. thaliana* or *S. lycopersicum* diets (Supplementary Fig S1). For the green module (*A. thaliana* diet) genera resulting in the highest kWithin values in the module were *Serratia* (15.135)*, Skermanella* (14.961), *Roseococcus* (14.955)*,* and *Variovorax* (14.874) (Table 5). In the brown module (*S. lycoperscium* diet*)* five genera expressed equal kWithin values (24.395); *Steroidobacter, Rubriviviax, Delftia, Sphingopysix,* and *Brevibacterium* (Table 5).

**Table 5:** Two of the eight WGCNA modules.

Module	Taxonomic hierarchy						Module membership value	kWithin value
	Kingdom	Phylum	Class	Order	Family	Genus		
Green	Bacteria	Proteobacteria	Gammaproteobacteria	Enterobacteriales	Enterobacteriaceae	*Serratia*	.9951	15.135
	Bacteria	Proteobacteria	Alphaproteobacteria	Rhodospirillales	Rhodospirillaceae	*Skermanella*	.9928	14.961
	Bacteria	Proteobacteria	Alphaproteobacteria	Rhodospirillales	Acetobacteracear	*Roseococcus*	.9930	14.955
	Bacteria	Proteobacteria	Gammaproteobacteria	Burkholderia	Comamonadaceae	*Variovorax*	.9939	14.874
Brown	Bacteria	Proteobacteria	Gammaproteo	xanthomonadales	Sinobacteraxeae	*Steroidobacter*	.9962	24.3945
	Bacteria	Proteobacteria	Betaproteobacteria	Burkholderiales	Comamonadaceae	*Rubriviviax*	.9962	24.3945
	Bacteria	Proteobacteria	Betaproteobacteria	Burkholderiales	Phyllobacteriaceae	*Delftia*	.9962	24.3945
	Bacteria	Proteobacteria	Alphaproteobacterial	Sphingomonadales	Sphingomonadaceae	*Sphingopyxis*	.9962	24.3945
	Bacteria	Actinobacteria	Actinobacteria	Actinomycetales	Brevibacteriaceae	*Brevibacterium*	.9962	24.3945

Displayed modules expressed the highest value of eigengenes and best describe and denote module members showing positive correlations to co-occurring genera detected within the same module. The green module indicates taxa within *Trichoplusia ni* gut communities after being fed with *A. thaliana* leaves. The brown module indicates taxa within *Trichoplusia ni* gut communities after being fed with *Solanum lycopersicum* leaves.

## Discussion

Generalist herbivores possess enzymatic tools (e.g., nitrogenases)^28,29^ that can aid in the conversion of toxic compounds and assist in digestion of their many available food sources. It has been recorded that many herbivores utilize epithelial tissue present within the midgut as a sensing organ^3,4^ and studies have shown insect herbivores converting cyanide to ammonia via endogenous nitrogenase production^3,4,28,29^. Such enzymatic tools stimulate the insect’s digestion of different plants by allowing the metabolization, detoxification, as well as uptake of an array of chemical compounds^30,31^. This capacity towards rapid metabolization of some chemical compounds may be potentiated if the insect population is kept on an exclusive diet, which is often the case for pest-populations living in monocultured agricultural systems^24^. Interestingly, the influence of the gut microbiota has not been deeply explored in the context of the generalist/polyphagous nature of insects. Although, the presence of specific microbiota in the gut has been shown to be involved in the adaptation of lepidopteran pests to different host diets and metabolites within those foods^22,31^. In our study the phyla Actinobacteria and Firmicutes were enriched in the population fed continuously with the artificial diet (13.07% and 12.06% respectively), compared to the populations fed with *S. lycopersicum* (9.91% to Actinobacteria; 14% to Firmicutes) and *A. thaliana* (8.56% to Actinobacteria; 7.73% to Firmicutes). The prevalence of the Actinobacteria and Firmicutes phyla across all populations may be attributed to a general symbiosis between these bacteria and *T. ni*, or the fact that insects used in this study came from one original population (obtained from Frontier Scientific, Inc.) that was fed and raised with the artificial diet prior to experimentation. Furthermore, we show that bacterial communities present on the plant leaves and in the artificial diet resulted in distinct phylogenetic clustering (p = 0.004) (Fig. 3a). The PCoA confirmed that each food or plant tissue source possessed unique resident microbial communities from one another.

Moreover, gut microbiota samples of insects fed with distinct diets resulted in significantly different communities (p = 0.035) visualized by PCoA clustering (Fig. 3b). These results may indicate that a modulation occurs in the structure and function of gut microbial communities within *T. ni* after feeding on exclusive diets. In our study, it was also observed that the artificial diet lowered the diversity within *T. ni* (Shannon index) compared to insect populations fed with plant-sourced diets (Table 3). In support of this observation, previous studies using both mice^32–34^ and human subjects^35,36^ suggest that an increase in species diversity or richness in gut microbiome systems can be attributed to diet source, along with the notion that these effects are observed to be strongest when subjects were fed with diets high in fruits and vegetables^32–36^.

We hypothesize that the insect diet can strongly influence the composition of its gut microbiota, and that in our study this colonization seemed to be functionally specific to the specific secondary metabolites present in each kind of plant tissue within diets administered in this study. Plant secondary metabolites (e.g., essential oils, alkaloids and phenolic compounds) have been shown to express antimicrobial properties against different bacterial families^22^. Furthermore, we propose that these plant derived compounds can act as inhibitors against members of the *T. ni* gut microbiome that are sensitive to the plant secondary metabolites from diets used in this study.

As such, here we interpret that a subset of bacteria detected in the guts of insects fed with each specific plant diet may be representative of organisms possessing abilities in detoxification of secondary metabolites produced by the plant used in the specific insect diet. The gut samples from populations fed with only *A. thaliana* leaves shared 19 genera with communities detected from leaves of *A. thaliana*, a member of the *Brassicaceae* family. Glucosinolates are prevalent in the *Brassicacea* plant family, and the catabolism of these compounds forms isothiocyanates which are used by plants to deter insect herbivory^37^. However, some specialist insects (*Brevicoryne brassicae* [Linnaeus, 1758]) have been shown to produce the glucosinolate-hydrolyzing myrosinase enzyme, although the true function of the *B. brassicae* (cabbage aphid) myrosinase remains undetermined^38^. Additionally, it has been reported that microbial myrosinases can also metabolize glucosinolates^39,40^. Thus, it is possible that the bacterial genera detected in both leaves of *A. thaliana* and the gut communities of insects fed with *A. thaliana* may be indicative of bacteria that express glucosinolate-toxicity-tolerance (or glucosinolate-degradation abilities). In support of this hypothesis, we observed that three genera (*Propionibacterium*, *Shinella* and *Terribacilus*) were exclusively more abundant in gut samples from insect populations fed with *A. thaliana* compared to those fed other diets. Members of the *Propionibacterium* genus have been reported to be tolerant to glucosinolate-toxicity^40–43^. In a recent study, an increase in the number of *Propionibacterium* colonies was observed in petri dishes supplemented with glucosinolate-compounds sourced from *A. thaliana* leaves^41^. Furthermore, we also observed that certain plant pathogenic genera reported to be susceptible to glucosinolate toxicity (e.g., members of *Xanthomoneacea*), were detected at lower relative abundances in *A. thaliana*-fed insects when compared to measures of these genera from gut samples of insects fed with *S. lycopersicum* or the artificial diet. Some bacterial species present in the guts of insects have been suggested as mediators of resistance to synthetic insecticides. The *Citrobacter* genus of the oriental fruit fly produces OPH-like enzymes to degrade the insecticide trichlorfon^44^. It was also shown that Arthrobacter and Pseudomonas isolated from insecticide-resistant *Spodoptera frugiperda* (J.E. Smith, 1797) larvae are highly effective in degrading deltamethrin and spinosyn^45^. Finally, the bacteria *Stenotrophomonas maltophilia* and *Enterococcus mundtii,* isolated from the intestine of the silkworm *Bombyx mori* (Linnaeus, 1758) were shown to influence resistance to the organophosphate insecticides^46,47^. Similarly in our study, the gut bacterial communities of the insects fed with leaves of *S. lycopersicum* shared 14 common genera with those detected on the leaves of *S. lycopersicum*. Among those microbes, *Agrobacterium* and *Rhizobium* groups were present in higher relative abundances (3.93% and 4.41%, respectively) when compared to the populations of *T. ni* fed with the other diets (Supplementary Table S2). These same genera were also more abundant in *S. lycopersicum* leaves when compared with *A. thaliana leaves* or the artificial diet (Supplementary Table S1). Both *Agrobacterium* and *Rhizobium* genera have been reported to degrade alkaloids^48,49^ as well as cyclic amines, often present in large quantities in many *Solanaceous* plant tissues^50,51^. As such, our study demonstrates potential adaptations in gut bacterial communities regarding gut-bacterial metabolism in pair with the degradation of compounds present within specific host diets. Curiously, the genus *Pseudomonas* had a lower relative abundance in gut samples from insects fed with *S. lycopersicum* leaves than in those fed with *A. thaliana* or the artificial diet (Supplementary Table S2). The *Pseudomonas* group contains members known for their pathogenic abilities towards a variety of invertebrates and humans^52,53^ and members of *Pseudomonas* expressed relatively lower abundance measures in insect populations fed with *S. lycopersicum.*

We further analyzed the microbiome data to pose speculations surrounding the predicted mechanisms utilized by bacteria to evade the toxicity of phytochemicals naturally produced within *A. thaliana* or *S. lycopersicum*. We found evidence of increased relative abundances of bacteria that produce phytochemical-degrading enzymes, or enzymes that aid in the degradation of the breakdown products of these phytochemicals. For instance, our PICRUSt analysis showed that bacteria present in insects fed with *A. thaliana* expressed increased relative abundance values of *alpha-L-rhamnosidase* (EC. 3.2.1.40). This enzyme has been reported to hydrolyze glycosidic substrates containing terminal alpha-L-rhamnose present in certain glucosinolates (e.g., glucomoringin)^25,54^. Additionally, it has been shown that *A. thaliana* contains genes involved in the biosynthesis of L-Rhamnose; thus, *A. thaliana* is likely able to produce glucosinolates with L-Rhamnose moieties^55^. As such, the presence of bacteria that produce *alpha-L-rhamnosidase* within insects fed *A. thaliana* may likely catabolize glucosinolates containing L-Rhamnose. Interestingly, enzymes coding for the thioglucosidase *myrosinase* (EC 3.2.1.147) were not observed to be present in insect gut samples analyzed. The limitation of PiCRUSt is that it relies on matching 16S sequences to related taxa that are fully sequenced for a survey of functional capacity. We note that xenobiotic degradation genes, like those for glucosinolate degradation, are typically plasma encoded and likely highly susceptible to horizontal gene transfer and would be hard to capture accurately with this approach.

For insects fed on a diet of *S. lycopersicum* leaves, the following enzymatic functions were observed to increase in relative abundances: alpha-amylase (EC.3.2.1.1), alpha-mannosidase (EC.3.2.1.24), beta-glucuronidase (EC.3.2.1.31), pullulanase (EC. 3.2.1.41), alpha-N-acetylglucoasminidase (EC.3.2.1.50), bifunctional sutolysin (EC.3.5.1.28 3.2.1.96) and mannosyl-glycoprotein endo-beta-N-acetylglucosaminidase (EC.3.2.1.96). All of the previous enzymes fall into the “glycosidases, or enzymes that hydrolyze O- and S-glycosyl compounds” category of the KEGG Enzymatic database^25^. *S. lycopersicum* is a *Solanaceous* crop known to produce glycoalkaloids^50^. It has been described that glycoalkaloids can readily undergo hydrolysis in the human stomach by bacterial glycosidases^56^. As such, in our study we show that predictive relative abundance values of many glycosidase-like enzymes were increased in gut microbiome samples from insects fed with leaves of *S. lycopersicum*.

A high proportion of bacteria that have not been previously described to degrade glucosinolates or glycol-alkaloids was also observed in the guts of insects fed with *A. thaliana* or *S. lycopersicum* such as *Pseudomonas, Streptococcus,* as well as several unclassified taxa (Supplementary Table S3). A Weighted Gene Co-expression Network Analysis was conducted to determine bacterial genera that could degrade those phytochemicals and allowed the survival and colonization of *Pseudomonas, Streptococcus* and others. Bacterial genera with the highest module membership and connectivity values (resulting from the WGCNA) are indicative of co-occurring genera in insect gut communities within each diet treatment. For example, the genus *Serratia* was highly correlated (kWithin: 15.135, Table 5) with the green module, which corresponds to gut microbiota from insects fed *A. thaliana*. *Serratia* members are resistant to the anti-microbial effects of isothiocyanates (degradation product of glucosinolates)^57^. Another genus that was highly correlated with the green module was *Variovorax* (kWithin: 14.874, Table 5)*,* which has been shown to proliferate in the presence of the glucosinolate goitrin^58^. Additional genera correlating with the green module were *Skermanella* (kWithin: 14.961, Table 5) and *Roseococcus* (kWithin: 14.955, Table 5). *Roseococcus*, a genus with members in the “purple non-sulfur bacteria” class (*R. thiosulfatophilus*), oxidizes thiosulfate (present in glucosinolates) when in the presence of an organic carbon substrate and aeration^59^. These bacterial genera showing high values of connectivity in the green module could potentially be providing environments that allow other bacteria to reside due the ability of these members to express some propensity of detoxification toward glucosinolates or their degradation products^42,43^.

The co-occurrence of bacterial taxa showing the highest module membership within the brown module indicated positive correlations between neighboring taxa in gut samples from insects fed with *S. lycopersicum.* The following genera displayed the same value for their kWithin Values (kWithin: 24.3945, Table 5): *Steroidobacter, Rubrivivax, Delftia, Sphingopyxis, and Brevibacterium*. These genera have also been reported to express some propensity to degrade alkaloids or associated aromatic ring structures typically contained within many alkaloids. *Steroidobacter* has been documented to degrade steroidal alkaloids such as those produced by *S. lycopersicum*^60^. Members of the genera *Rubrivivax* and *Delftia* have been observed to degrade aromatic hydrocarbons or benzene ring structures associated with many alkaloids^61,62^. For the genus *Sphingopyxis*, reports have demonstrated that members can effectively breakdown antimicrobial alkaloids such as berberine^63^. Lastly, the genus *Brevibacterium* can effectively degrade the antimicrobial and N-containing pyridine alkaloids^64^. Similar to the bacteria within insects fed with *A. thaliana,* we speculate that these aforementioned genera may have allowed for colonization of other bacteria in the gut of insects fed with *S. lycoperscium* leaves due to their glycoalkaloid detoxification abilities. Further metagenomics and food studies are needed to corroborate these hypotheses.

Generalist species of insects have a competitive advantage over other pests in regard to available food choices. Insects with many available food choices have greater survival advantages in an ecosystem where some food sources are easily depleted^65^. In this study, we showed that the gut microbiome composition of polyphagous insects can change based on the plant diet, that these microbiota are likely acquired from the diets themselves, and that this adaptability may be related to the ability of certain microbes in the detoxification of antimicrobial phytochemicals. Our findings may allow for further experimentation of insect diet preference using neonate larvae, in addition to the incorporation of new methodologies to deal with generalist insects in agriculture.

Finally, there are examples in the literature in which the disruption of the associations between microbes and their hosts lead to negative consequences for the insects^67–68,71^. For example, in aphids the modification of the body color provided by facultative symbiotic microorganisms can determine the susceptibility of these insects to predation or parasitism^66^. As such the results of our study could lead to novel Integrated Pest Management (IPM) strategies targeted to the insect’s gut microbiota to disrupt their ability to detoxify plant-toxins present in the crop diet.

## Methods

### Insect growth conditions

An initial colony of *T. ni* larvae in the third instar was obtained from Frontier Agricultural Sciences Inc. (Newark, DE), a company that sells, maintains and feeds uniform insect colonies using an artificial diet without the addition of antibiotics. The larvae were then divided into three groups; each population was composed of twenty individuals and kept in sterile Magenta boxes. Each Magenta box contained an average of five larvae. The Magenta boxes were kept on shelves and covered with Nylon screens that prevented the insects from escaping.

The larvae in each treatment were fed daily with *S. lycopersicum* leaves, *A. thaliana* leaves or with 4 g portions of the artificial diet cubes which consisted of a protein base made with soy flour and wheat germ (Item Code #: F9772 by Frontier Agricultural Sciences Inc). Larvae in each treatment were kept at room temperature (26 °C), 75% humidity with cycles of 16 h light, 8 h dark photoperiod conditions. When the larvae reached the pupae stage, they were transferred to clear plastic containers (height 26 cm, upper diameter 10.5 cm, lower diameter 13 cm) covered with insect-proof mesh cloth and sealed with rubber bands, until they reached the moth stage. In the plastic containers, the moths were fed daily using filter paper soaked with 100 µl (per plastic bottle) of a 9:1 Milli-Q water: honey solution. The female and male moths in the plastic containers were allowed free crossing to mimic natural breeding cycles. The eggs were laid by the moths on clean filter papers and kept inside each container. Eggs deposited on the filter papers were transferred to pre-sterilized Magenta boxes until the moment of hatching. After the emergence of first instar *T. ni* larvae in the Magenta boxes, *A. thaliana* leaves, *S. lycopersicum* leaves or artificial diet cubes were delivered daily to insects in each treatment group. The larvae of each subsequent generation were fed accordingly to match the diet of those larvae that served as the progenitors.

### Plant growth conditions

*A. thaliana* ecotype (Col-0) seeds were purchased from Lehle Seeds (Round Rock, TX, USA). The seeds were surface sterilized in 2.0% sodium hypochlorite for 2 min, followed by three washes with sterile distilled water. The surface-sterilized seeds were placed directly on fibrous peat moss Promix Bx substrate mixed with vermiculite (1:1) for germination. The plants were grown in plastic trays and incubated in a growth chamber. A photoperiod of 16 h light and 8 h dark at 25 ± 2 °C was maintained for growing conditions. After growing *A. thaliana* for 15 days, leaves and stems were used as food for the insect populations. *S. lycopersicum* (cv. Rutgers) seeds were purchased from Burpee Seeds (Warminster, PA, USA). The seeds were surface sterilized in 2.0% sodium hypochlorite for 2 min, followed by three washes with sterile distilled water. Surface-sterilized seeds were placed directly on the fibrous peat moss Promix Bx substrate mixed with vermiculite (1:1) for germination and grown in plastic tray contained within a growth chamber. A photoperiod of 16 h light and 8 h dark at 28 ± 2 °C was kept for growing conditions. *S. lycopersicum* was grown for 30 days before excision of leaves and stems to serve as food for the insect populations.

### Food preference trial

After three complete life cycles of *T. ni* feeding on one particular diet, the populations were separated according to diet, and insects were exposed to a food preference test with a choice between the three different food sources using the methodology described by Raffa et al*.*^69^. In the choice trial, twelve replicates were used for each population, and each replicate was composed of fourth instar larvae. The larvae were devoid of food for 24 h before initiating the choice study. The larvae were arranged in a Petri dish (140 mm diameter) and lined with moistened filter paper to maintain humidity. Three Eppendorf tubes (size 2 ml) were placed in the Petri dishes; each tube was filled separately with pieces of *S. lycopersicum* leaves, fragments of *A. thaliana* leaves or parts of the artificial diet.

All three tubes containing food were weighed before the start of the experiment and again after 24 h to determine how many milligrams each larva had consumed from each diet during the 24 h period. The difference in weight of the specific diets at 0 h and 24 h was considered the measurement of choice in this study. It should be noted that some larvae fed on more than one diet during the 24 h period. Each set of plates with all treatments were arranged randomly. The trays were kept in growth chambers (*Percival Scientific*) regulated at 25 °C ± 1 °C, 70 ± 10% relative humidity, and 16 h of light. Difference in weight of the diets for each treatment indicated the insect’s affinity to eat the same diet as their progenitors. For this test, the diet that was consumed the most, or experienced the highest reduction in mass after 24 h, was used as an indicator to determine the insect’s food preference following the methodology proposed by Raffa et al*.*^69^ for determination of food preference in insect models.

After 24 h the trays were removed from the growth chambers and weighed. *T. ni* larvae were also weighed before experimentation and again after 24 h to verify if there was weight gain among the three different populations tested. The experiment was repeated twice. The data of all the tests were subjected to ANOVA with Tukey’s post hoc test, at the 5% probability level. The free software *Past* and *Sisvar* were used for the statistical analyses and software *Origin 8.5* for the creation of Figs. 1 and 2.

### DNA extraction

#### Insect gut DNA extraction

Samples were obtained from larvae in the fourth developmental instar stage; after three complete life cycles of each population. Each gut was removed with sterile tweezers, and the gut tissue and inner contents were then homogenized by shaking in a sterile tube containing sterile glass beads (0.5 mm diameter) and 0.5 ml of PBS buffer (pH 7.5) for 15 min using a Vortex. Intestines of 20 larvae were combined per group and extracted using the Qiagen DNeasy PowerSoil kit (Hilden, Germany) according to the manufacturer's instructions. A total of three replicates per diet group were obtained. The DNA concentration was measured by a Nanodrop spectrophotometer (*Thermo Fisher Scientific*). The extracted DNA was stored at − 20 °C for further analysis. Insects from generation four of this study (n = 80 per diet treatment) were separated and used for DNA extraction (n = 60) as well as the food preference trial (n = 20).

#### Plant and artificial diet DNA extraction

Three replicates of each diet were used for DNA extraction from plant leaves (*A. thaliana or S. lycopersicum*) or the artificial diet in order to compare the bacterial communities found in each diet to those found in the gut of *T. ni*. Plants and insects were cultured separately. The vegetal diets (plant leaves) and the artificial diet used for the DNA extraction were collected directly from plants grown in growth chambers or from the artificial diet tray, without allowing any insect contact. Each sample was composed of 50 mg of plant leaves or of the artificial diet according to the experimental group to which it belonged. The total DNA was extracted using the MoBio PowerPlant DNA isolation kit (MoBio purchased by Qiagen in 2016) according to the manufacturer's instructions. The DNA concentration was measured by a Nanodrop spectrophotometer (*Thermo Fisher Scientific*). The extracted DNA was stored at − 20 °C for downstream sequencing analysis.

## Illumina MiSeq Sequencing of the Bacterial 16S rRNA gene

### The PCR amplification for Illumina Miseq

The sequencing of all gut samples was performed according to standard protocols devised by Illumina. The V3-V4 hypervariable region of the 16S rRNA gene was targeted using a quantitative polymerase chain reaction (PCR) to characterize and estimate bacterial communities present in the DNA samples. Briefly, a first round of PCR using a modified version of primer set 341F/785R (F5′–TCGTCGGCAGCGTCAGATGTGTATAAGAGACAGCCTACGGGAGGCAGCAG-3′) and (R5′- GTCTCGTGGGCTCGGAGATGTGTATAAGAGACAGGACTACHVGGGTATCTAATCC-3′) was conducted to include Illumina MiSeq adapters, with adapter sequences denoted by underlining^70^.

An additional round of PCR was then performed in 20 µl reaction volumes containing 2 µl of template DNA (5 ng/ µl) along with 18 µl of the master mix. The master mix consisted of 10 µL of 2X Maxima SYBR Green (Thermo Scientific, Waltham, MA, USA), and 1 µL each (10 µM) of forward and reverse primers, along with 6 µL of molecular grade water. The PCR thermal cycling conditions were as follows: 95 °C for 5 min, 25 cycles of 95 °C for 40 s, 55 °C for 30 s, 72 °C for 60 s, and a final step of extension at 72 °C for 5 min. The resulting amplicons were purified using an in-house preparation of solid phase reversible immobilization (SPRI) magnetic beads based on the modifications of Glenn (2011)^71^ and original protocol of Rohland and Reich (2012)^72^.

### Miseq library preparation

The MiSeq library was prepared by standard library construction protocol as detailed in the "16S metagenomics sequencing library preparation procedures". Available at https://support.illumina.com/downloads/16s_metagenomic_sequencing_library_preparation.htm. A second round of PCR was used to attach unique Illumina Nextera XT index sequences was performed according to the methods of Li et al., (2019)^73^. The final quality of the DNA library (amplicon size and purity) was checked and quantified using the Agilent 4200 Tapestation at CSU’s Next Generation Sequencing Laboratory (Fort Collins, CO) and Kapa Biosystems (Sigma-Aldrich, St Louis, MO, USA) qPCR was preformed according to the manufactures instructions to confirm concentration of the library. The final pooled sample was diluted to 4 nM and the library was denatured using 0.2 NaOH, diluted to 10 pM using provided HT1 buffer, and spiked with 15% PhiX standard diversity control. Illuminas MiSeq v3 600-cycle reagent kit (Illumina, San Diego, USA) was used for library dilution and loading onto the MiSeq at CSU’s Next Generation Sequencing Laboratory (Fort Collins, CO, USA).

### Bacterial 16S rRNA gene sequence analysis

De-multiplexed raw FASTQ files were processed with the DADA2 pipeline^74^. Briefly, all primers were removed from each sequence using open-source Python program Cutadapt^75^, amplicon sequence variants were inferred with the default pipeline in DADA2. Sequence variants identified with DADA2 were classified using the GreenGenes reference database (v_13_5_99) at a threshold of 97% similarity. All downstream analyses were conducted using the software myPhyloDB v.1.2.0^76^ or Microbiome Analyst^78^. Shannon Diversity index was calculated using the phyloseq R package and was used to assess initial bacterial alpha diversity and was reported based on the number of different phyla within a treatment. For beta diversity, microbial community composition was analyzed using principal coordinates analysis (PCoA) based on the Bray–Curtis’ dissimilarity index. A complementary non-parametric multivariate statistical test, including permutational analysis of variance (perMANOVA) and non-parametric univariate ANcOVA analyses, were used to test the differences in microbial communities with the Bray–Curtis distance and 999 permutations with myPhyloDB in addition to figure generation^76^.

A total of 1,854,660 sequence reads were obtained resulting in an average of 102,592 reads per sample. Each resulting taxonomic profile was used to determine both bacterial phyla-, family-, genera- and/or gene-specific relative abundance measures. Gene-specific bacterial abundances are based on a comprehensive list of functions associated with bacteria. The selected genes analyzed from the KEGG database^26^ incorporate processes such as alkaloid and glucosinolate degradations. The taxonomic mapping and phylogenetic reconstruction necessary to identify populations of each gene-specific abundance was performed using myPhyloDB’s implementation of PICRUSt^77^. Weighted correlation network analyses were conducted using myPhyloDB’s implementation of the R package WGCNA^27^. For each genus described, module membership values were calculated using the WGCNA function, correlating module eigengene with gene expression values. The kWithin values indicate the connectivity of each genus within a single module, to all other co-occurring genera within the same module.

A linear discriminant effect size (LEfSe) analysis was implemented using the Microbiome Analyst^78^ open source software in order to identify bacterial taxa that were significantly associated with each treatment, a threshold alpha value of 0.05 for the LSmeans & Tukey's HSD post-hoc test was implemented. The ASV (Amplicon Sequence Variants) table resulting as output from myPhyloDB was then used to verify detected ASVs at the family level shared between each experimental group. The shared and unique ASVs among treatments were counted, and their distributions are shown in a Venn diagram with the *'jvenn'* that is a plugin for the ‘*jQuery’* Javascript library [79]. The tables with the shared and unique ASVs among treatments at the family level were prepared with a tool that may be found at URL: http://bioinformatics.psb.ugent.be/webtools/Venn/.

## Supplementary Information

Supplementary Information
