# Supplementary material for: The gut microbiota composition of Trichoplusia ni is altered by diet and may influence its polyphagous behavior
Source: Sci Rep. 2021 Mar 11;11:5786. doi: 10.1038/s41598-021-85057-0 (PMC7970945; doi:10.1038/s41598-021-85057-0)
Supplement: Supplementary file 1 — Supplementary Information [file 41598_2021_85057_MOESM1_ESM.docx]

The gut microbiota composition of *Trichoplusia ni* is altered by diet and may influence its polyphagous behavior

M. Leite-Mondin^1,4+^; M. J. DiLegge^4+^; D.K. Manter^2^; T.L. Weir^3^; M. C. Silva-Filho^1^; J. M. Vivanco^4*^

^1^Departmento de Genética, Escola Superior de Agricultura Luiz de Queiroz, Universidade de São Paulo, Av. Pádua Dias, 11, 13418-900, Piracicaba, SP, Brazil

^2^USDA, Center for Agricultural Research Services, Soil-Plant-Nutrient Research Unit, Fort Collins-CO, USA

^3^Department of Food Science and Human Nutrition, Colorado State University, Fort Collins-CO, USA

^4^Department of Horticulture and Landscape Architecture, Colorado State University, Fort Collins-CO, USA

* [j.vivanco@colostate.edu](mailto:j.vivanco@colostate.edu)

^+^Both authors contributed equally

**Supplementary Material**

**Supplemental Figure S1.**: Box plots showing the correlation (pos/neg), of the co-occurring taxa in *T. ni* gut grouped within each module. Positive or negative values indicate the correlation of the co-occurring taxa in each module to influence co-occurring/neighboring taxa, for each treatment of differentially administered insect diets. Brown (Supplementary Figure 1a) module positively correlated with taxa occurring from insects fed with *S. lycoperscium* leaves. Green (Supplementary Figure 1b) module positively correlated with taxa occurring from insects fed with *Arabidopsis* leaves.


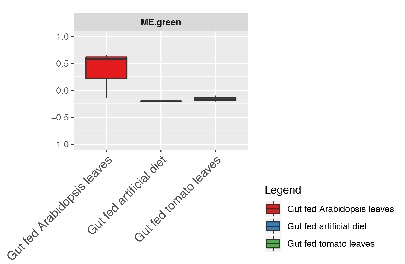

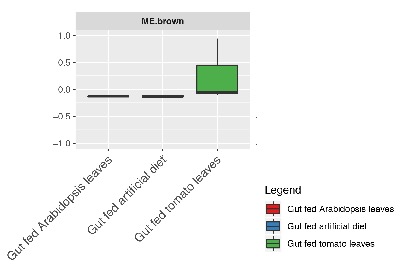


**(a)**

**(b)**

**Supplemental Figure S2:** Dendrogram. Weighted correlation network analysis (WGCNA) of gut bacteria communities from three treatments of differentially fed insect populations (*n*=3) of *Trichoplusia ni*. Bacterial phylotypes (genera) were clustered by total abundance (16S rRNA copies/g soil) as shown by the dendrogram and correlation heat map. Clusters of co-occurring phylotypes or modules are indicated by the color bars. The intensity of red coloring in the heat map indicates the strength of the correlation between pairs of modules that group the phylotypes.


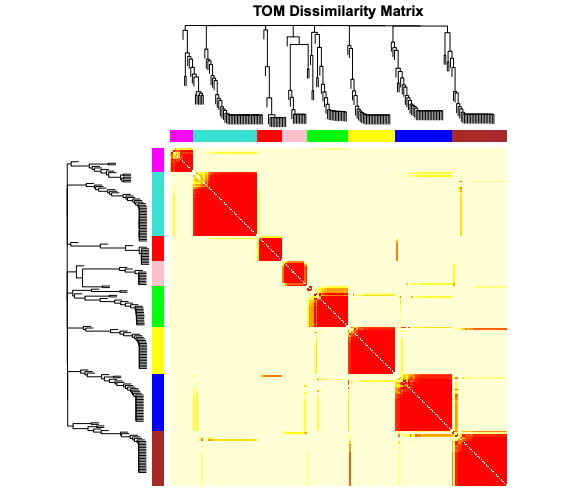


| **Supplemental table S1:** Percentage for bacterial ASVs grouped by the highest-classified taxonomic level for relative abundance in the samples from different diets/food. | | | | |
| --- | --- | --- | --- | --- |
| *Name* | *Taxa level* | *A.thaliana (%)* | *S. lycopersicum (%)* | *Artificial Diet (%)* |
| *Pseudomonas** | genus | 0.1232 | 0.0529 | 2.6021 |
| *Streptomyces** | genus | 0.077 | 0 | 0 |
| *Shinella* | genus | 0.0343 | 0 | 0 |
| *Rhodoplanes* | genus | 0.0303 | 0 | 0.0432 |
| *Mesorhizobium* | genus | 0.0056 | 0 | 0.0178 |
| *Dokdonella* | genus | 0.0039 | 0 | 0 |
| *Cellvibrio* | genus | 0.0034 | 0 | 0 |
| *Sphingopyxis* | genus | 0.0033 | 0 | 0 |
| *Methylibium* | genus | 0.0033 | 0 | 0 |
| *Propionibacterium** | genus | 0.0025 | 0 | 0.0485 |
| *Acinetobacter* | genus | 0.002 | 0 | 0 |
| *Flavobacterium* | genus | 0.0014 | 0 | 0 |
| *Agrobacterium** | genus | 0 | 0.1594 | 0 |
| *Sphingobium** | genus | 0 | 0.0353 | 0 |
| *Sphingomonas* | genus | 0 | 0.0196 | 0 |
| *Delftia** | genus | 0 | 0.0123 | 0 |
| *Rhizobium* | genus | 0 | 0.012 | 0.0106 |
| *Azospirillum** | genus | 0 | 0.008 | 0 |
| *Chryseobacterium* | genus | 0 | 0.0057 | 0.0091 |
| *Limnohabitans* | genus | 0 | 0.0051 | 0 |
| *Sphingobacterium* | genus | 0 | 0.0029 | 0.0049 |
| *Methylotenera* | genus | 0 | 0.0023 | 0 |
| *Bacillus* | genus | 0 | 0 | 0.0421 |
| *Staphylococcus* | genus | 0 | 0 | 0.0348 |
| *Corynebacterium* | genus | 0 | 0 | 0.0218 |
| *Exiguobacterium* | genus | 0 | 0 | 0.0218 |
| *Plesiocystis* | genus | 0 | 0 | 0.0216 |
| *Paenibacillus* | genus | 0 | 0 | 0.021 |
| *Lentzea* | genus | 0 | 0 | 0.0183 |
| *Geobacillus* | genus | 0 | 0 | 0.0158 |
| *Brevibacillus* | genus | 0 | 0 | 0.0139 |
| *Brachybacterium* | genus | 0 | 0 | 0.0136 |
| *Actinobaculum* | genus | 0 | 0 | 0.0134 |
| *Actinomadura* | genus | 0 | 0 | 0.013 |
| *Planctomyces* | genus | 0 | 0 | 0.0116 |
| *Rhodanobacter* | genus | 0 | 0 | 0.0106 |
| *Serratia* | genus | 0 | 0 | 0.0077 |
| *Schlegelella* | genus | 0 | 0 | 0.007 |
| *Clostridium* | genus | 0 | 0 | 0.0066 |
| *HTCC* | genus | 0 | 0 | 0.0055 |
| *Aeromicrobium* | genus | 0 | 0 | 0.0054 |
| *Acinetobacter* | genus | 0 | 0 | 0.0049 |
| *mitochondria** | family | 6.3115 | 11.287 | 36.197 |
| *Moraxellaceae** | family | 0.1494 | 0.1016 | 4.2123 |
| *Enterobacteriaceae** | family | 0.0152 | 0.2022 | 0.0115 |
| *Xanthomonadaceae* | family | 0.0119 | 0.0056 | 0.0061 |
| *Rhizobiaceae* | family | 0.009 | 0 | 0 |
| *Nitrosomonadaceae* | family | 0.0083 | 0 | 0 |
| *Phyllobacteriaceae* | family | 0.0071 | 0 | 0 |
| *Rhodospirillaceae* | family | 0.0067 | 0 | 0 |
| *OM27* | family | 0.0057 | 0 | 0 |
| *Bradyrhizobiaceae* | family | 0.0048 | 0 | 0 |
| *Hyphomicrobiaceae* | family | 0.0047 | 0 | 0 |
| *Oxalobacteraceae* | family | 0.0034 | 0 | 0 |
| *Methylophilaceae* | family | 0.0029 | 0 | 0 |
| *Nocardioidaceae* | family | 0.0027 | 0 | 0.0095 |
| *Caulobacteraceae** | family | 0 | 0.0636 | 0 |
| *Comamonadaceae* | family | 0 | 0.0102 | 0 |
| *AKIW874* | family | 0 | 0 | 0.0165 |
| *Streptomycetaceae* | family | 0 | 0 | 0.0121 |
| *Beijerinckiaceae* | family | 0 | 0 | 0.0099 |
| *Peptostreptococcaceae* | family | 0 | 0 | 0.0094 |
| *AK1AB1_02E* | family | 0 | 0 | 0.0086 |
| *Gaiellaceae* | family | 0 | 0 | 0.0065 |
| *Rhodocyclaceae* | family | 0 | 0 | 0.0064 |
| *Streptophyta** | order | 92.753 | 87.314 | 54.72 |
| *Rhodospirillales* | order | 0.0046 | 0 | 0 |
| *Rhizobiales* | order | 0.0037 | 0 | 0 |
| *MND1* | order | 0.0033 | 0 | 0 |
| *Ellin329* | order | 0.0018 | 0 | 0 |
| *SC-I-84* | order | 0.0008 | 0 | 0 |
| *JG30-KF-CM45* | order | 0 | 0 | 0.0178 |
| *Ellin6067* | order | 0 | 0 | 0.0117 |
| *Sphingomonadales* | order | 0 | 0 | 0.0073 |
| *Gemmatimonadetes* | class | 0 | 0 | 0.0046 |
| *Unclassified** | Unclassified | 0.3995 | 0.7006 | 1.6972 |

| *ANCOVA univariate test (P<0.05) |  |  |  |  |  |
| --- | --- | --- | --- | --- | --- |
| **Supplementary table S2:** Percentage for bacterial ASVs grouped by highest classified taxonomic level for relative abundance in the samples from gut microbiome to each population of *T. ni* fed with an exclusive *diet*. | | | | | |
|  |  |  |  |  | |
| *Name* | *Taxa level* | *Population fed with* | *Population fed with* | *Population fed with Artificial Diet (%)* | |
|  |  | *A. thaliana leaves (%)* | *S. lycopersicum leaves (%)* |  |  |
| *Serratia* | genus | 22.925 | 1.3234 | 0 | |
| *Pseudomonas* | genus | 10.981 | 9.2787 | 15.769 | |
| *Propionibacterium* | genus | 4.2248 | 4.0474 | 3.4434 | |
| *Shinella** | genus | 1.6956 | 0.2046 | 0 | |
| *Terribacillus* | genus | 1.349 | 0 | 0 | |
| *Streptococcus* | genus | 1.0683 | 6.8859 | 3.199 | |
| *Novosphingobium* | genus | 0.8776 | 0 | 0 | |
| *Microbacterium** | genus | 0.8237 | 0.0557 | 0.0881 | |
| *Bacillus* | genus | 0.7813 | 3.3365 | 0.1843 | |
| *Stenotrophomonas* | genus | 0.7788 | 0 | 0 | |
| *Arthrobacter* | genus | 0.5107 | 0.075 | 0 | |
| *Corynebacterium* | genus | 0.4849 | 0.743 | 0.4625 | |
| *Agrobacterium* | genus | 0.4734 | 3.9317 | 0.0924 | |
| *Skermanella* | genus | 0.4062 | 0 | 0 | |
| *Mesorhizobium* | genus | 0.3252 | 0 | 0 | |
| *Ochrobactrum* | genus | 0.2708 | 0 | 0 | |
| *Staphylococcus* | genus | 0.2558 | 0.7664 | 0.607 | |
| *Deinococcus* | genus | 0.2438 | 0.056 | 0.0237 | |
| *Paracoccus* | genus | 0.2427 | 0.242 | 0.1307 | |
| *Roseococcus* | genus | 0.2281 | 0 | 0 | |
| *Sphingobium* | genus | 0.1529 | 0 | 0 | |
| *Pseudonocardia* | genus | 0.1398 | 0.0828 | 0 | |
| *Actinobaculum* | genus | 0.1332 | 0.3456 | 0.3125 | |
| *Haemophilus* | genus | 0.1315 | 0.0517 | 0.0139 | |
| *Rhodococcus* | genus | 0.1305 | 0.044 | 0.0823 | |
| *Cryocola* | genus | 0.1285 | 0 | 0 | |
| *Nocardioides* | genus | 0.1249 | 0.1946 | 0.0164 | |
| *Methylobacterium* | genus | 0.1201 | 0 | 0 | |
| *Sphingomonas* | genus | 0.1164 | 0.3061 | 0.3028 | |
| *Lactobacillus* | genus | 0.1091 | 0.0316 | 0 | |
| *Rhodoplanes* | genus | 0.1055 | 0.2115 | 0 | |
| *Variovorax* | genus | 0.1054 | 0 | 0 | |
| *Friedmanniella* | genus | 0.1035 | 0.0241 | 0 | |
| *Enhydrobacter* | genus | 0.096 | 0.0472 | 0 | |
| *Acinetobacter* | genus | 0.0897 | 0.3447 | 0.0008 | |
| *Lactococcus* | genus | 0.0828 | 0 | 0.0939 | |
| *Janibacter* | genus | 0.0783 | 0 | 0 | |
| *Mycobacterium* | genus | 0.0771 | 0.3871 | 0.4187 | |
| *Enterococcus** | genus | 0.0699 | 0 | 1.9556 | |
| *Streptomyces* | genus | 0.0628 | 1.0424 | 5.2551 | |
| *Kocuria* | genus | 0.0578 | 0 | 0 | |
| *Glycomyces* | genus | 0.0506 | 0 | 0 | |
| *Hymenobacter* | genus | 0.0485 | 0.0476 | 0 | |
| *Hyphomicrobium* | genus | 0.0419 | 0 | 0 | |
| *Moryella* | genus | 0.0378 | 0.0485 | 0 | |
| *Devosia* | genus | 0.0349 | 0.3424 | 0 | |
| *Kaistia* | genus | 0.0329 | 0 | 0 | |
| *Cellulomonas* | genus | 0.0321 | 0.0723 | 0 | |
| *Eikenella* | genus | 0.0196 | 0.084 | 0.0023 | |
| *Paenibacillus* | genus | 0.0148 | 0 | 0 | |
| *Tepidimonas* | genus | 0.0119 | 0 | 0 | |
| *Sporomusa* | genus | 0.0044 | 0 | 0 | |
| *Rhizobium* | genus | 0 | 4.4178 | 0 | |
| *Rothia* | genus | 0 | 1.2889 | 0.3302 | |
| *Luteimonas* | genus | 0 | 0.4915 | 0 | |
| *Veillonella** | genus | 0 | 0.3304 | 0.0286 | |
| *Methylobacterium* | genus | 0 | 0.2514 | 0 | |
| *Gemella* | genus | 0 | 0.1943 | 0.3954 | |
| *Anaerococcus* | genus | 0 | 0.1911 | 0 | |
| *Jeotgalicoccus* | genus | 0 | 0.1498 | 0 | |
| *Dokdonella* | genus | 0 | 0.1323 | 0 | |
| *Candidatus_Solibacter* | genus | 0 | 0.1308 | 0 | |
| *Actinomyces* | genus | 0 | 0.1299 | 0 | |
| *Cellulosimicrobium* | genus | 0 | 0.1261 | 0 | |
| *Labrys* | genus | 0 | 0.1246 | 0 | |
| *Dermacoccus* | genus | 0 | 0.1138 | 0 | |
| *Granulicatella* | genus | 0 | 0.1055 | 0.03 | |
| *Exiguobacterium* | genus | 0 | 0.1007 | 0 | |
| *Burkholderia* | genus | 0 | 0.0985 | 0 | |
| *Aggregatibacter* | genus | 0 | 0.0845 | 0 | |
| *Kaistobacter* | genus | 0 | 0.0746 | 0.1361 | |
| *Acetobacter* | genus | 0 | 0.073 | 0 | |
| *Leuconostoc* | genus | 0 | 0.0651 | 0 | |
| *Asticcacaulis* | genus | 0 | 0.0628 | 0 | |
| *Finegoldia* | genus | 0 | 0.0579 | 0 | |
| *Solibacillus* | genus | 0 | 0.057 | 0 | |
| *Chryseobacterium* | genus | 0 | 0.0563 | 0 | |
| *Rhodanobacter* | genus | 0 | 0.0561 | 0.5192 | |
| *Mycoplana* | genus | 0 | 0.0559 | 0 | |
| *Prevotella* | genus | 0 | 0.0545 | 0.1144 | |
| *Facklamia* | genus | 0 | 0.0485 | 0.2965 | |
| *Sphingopyxis* | genus | 0 | 0.0467 | 0 | |
| *Thermomonas* | genus | 0 | 0.0465 | 0 | |
| *Actinocatenispora* | genus | 0 | 0.0398 | 0 | |
| *Paludibacter* | genus | 0 | 0.0387 | 0 | |
| *Steroidobacter* | genus | 0 | 0.0379 | 0 | |
| *Dermabacter* | genus | 0 | 0.0302 | 0 | |
| *Clavibacter* | genus | 0 | 0.0282 | 0 | |
| *Denitrobacter* | genus | 0 | 0.0278 | 0 | |
| *Amaricoccus* | genus | 0 | 0.0276 | 0 | |
| *Roseomonas* | genus | 0 | 0.0275 | 0 | |
| *Tetrathiobacter* | genus | 0 | 0.0275 | 0 | |
| *Mesorhizobium** | genus | 0 | 0.0253 | 0 | |
| *Lautropia* | genus | 0 | 0.0203 | 0 | |
| *Brevibacterium* | genus | 0 | 0.0195 | 0 | |
| *Porphyromonas* | genus | 0 | 0.0175 | 0 | |
| *Rubrivivax* | genus | 0 | 0.0171 | 0 | |
| *Amycolatopsis* | genus | 0 | 0.0162 | 0 | |
| *Flavobacterium* | genus | 0 | 0.0146 | 0.0649 | |
| *Delftia* | genus | 0 | 0.0143 | 0 | |
| *Shewanella* | genus | 0 | 0.0115 | 0 | |
| *Peptoniphilus* | genus | 0 | 0.0105 | 0 | |
| *Brevundimonas* | genus | 0 | 0.0013 | 0 | |
| *Achromobacter** | genus | 0 | 0 | 0.3156 | |
| *Blastococcus* | genus | 0 | 0 | 0.1878 | |
| *Limnohabitans* | genus | 0 | 0 | 0.1538 | |
| *Balneimonas* | genus | 0 | 0 | 0.1188 | |
| *Gluconobacter* | genus | 0 | 0 | 0.1138 | |
| *Clavibacter* | genus | 0 | 0 | 0.1066 | |
| *Rathayibacter* | genus | 0 | 0 | 0.0922 | |
| *Erwinia* | genus | 0 | 0 | 0.0875 | |
| *Geobacillus* | genus | 0 | 0 | 0.0808 | |
| *Couchioplanes* | genus | 0 | 0 | 0.0788 | |
| *Salinicoccus* | genus | 0 | 0 | 0.0731 | |
| *Cellvibrio* | genus | 0 | 0 | 0.0712 | |
| *Luteolibacter* | genus | 0 | 0 | 0.0502 | |
| *Flavisolibacter* | genus | 0 | 0 | 0.0469 | |
| *Agrococcus* | genus | 0 | 0 | 0.0437 | |
| *Oribacterium* | genus | 0 | 0 | 0.0354 | |
| *Ramlibacter* | genus | 0 | 0 | 0.0331 | |
| *Dietzia* | genus | 0 | 0 | 0.0318 | |
| *Dyella* | genus | 0 | 0 | 0.0193 | |
| *Actinomadura* | genus | 0 | 0 | 0.0093 | |
| *Lysobacter* | genus | 0 | 0 | 0.0046 | |
| *Aeromicrobium* | genus | 0 | 0 | 0 | |
| *Moraxellaceae* | family | 17.801 | 14.419 | 24.854 | |
| *Enterobacteriaceae* | family | 3.8221 | 20.8 | 13.036 | |
| *Planococcaceae** | family | 3.2553 | 0 | 4.8961 | |
| *Xenococcaceae* | family | 2.7706 | 0.0382 | 0 | |
| *Sphingomonadaceae* | family | 1.9033 | 0 | 0 | |
| *Erythrobacteraceae* | family | 0.4412 | 0.0571 | 0.0407 | |
| *mitochondria* | family | 0.3769 | 4.44 | 0 | |
| *Rhizobiaceae* | family | 0.3584 | 0.0602 | 0 | |
| *C111* | family | 0.2965 | 0.036 | 0 | |
| *Micrococcaceae* | family | 0.2717 | 0.0876 | 0.5461 | |
| *Hyphomicrobiaceae* | family | 0.22 | 0 | 0 | |
| *Alcaligenaceae** | family | 0.1804 | 0 | 0 | |
| *Rhodospirillaceae* | family | 0.1633 | 0.3075 | 0.2929 | |
| *Gaiellaceae* | family | 0.1627 | 0.0137 | 0.0909 | |
| *Nocardioidaceae** | family | 0.1595 | 0.3921 | 0.0344 | |
| *Bradyrhizobiaceae* | family | 0.1503 | 0.2656 | 0.0107 | |
| *Pseudomonadaceae* | family | 0.0972 | 0.1239 | 0.1147 | |
| *Oxalobacteraceae* | family | 0.087 | 0.1197 | 0.279 | |
| *Promicromonosporaceae* | family | 0.0837 | 0 | 0 | |
| *Microbacteriaceae* | family | 0.0753 | 0.0856 | 0.1231 | |
| *Frankiaceae* | family | 0.0707 | 0 | 0 | |
| *RFP12* | family | 0.0531 | 0 | 0 | |
| *Intrasporangiaceae* | family | 0.0439 | 0.08 | 0 | |
| *Comamonadaceae** | family | 0.0337 | 0.0828 | 0.0019 | |
| *Bacillaceae* | family | 0.0315 | 0.0632 | 0 | |
| *Neisseriaceae* | family | 0.0306 | 0.0576 | 0.083 | |
| *Weeksellaceae* | family | 0.0268 | 0.0136 | 0 | |
| *Conexibacteraceae* | family | 0.0266 | 0 | 0 | |
| *Pirellulaceae* | family | 0.0045 | 0 | 0 | |
| *Streptococcaceae* | family | 0 | 1.3994 | 0 | |
| *Solirubrobacteraceae* | family | 0 | 0.2069 | 0.0158 | |
| *Caulobacteraceae* | family | 0 | 0.1939 | 0.0047 | |
| *Halomonadaceae* | family | 0 | 0.1445 | 0 | |
| *Acetobacteraceae* | family | 0 | 0.1287 | 0 | |
| *Methylobacteriaceae* | family | 0 | 0.1009 | 0 | |
| *Sphingomonadaceae* | family | 0 | 0.0928 | 0 | |
| *Alicyclobacillaceae* | family | 0 | 0.0693 | 0 | |
| *Streptomycetaceae** | family | 0 | 0.0669 | 0.4823 | |
| *Geodermatophilaceae* | family | 0 | 0.0443 | 0.1018 | |
| *0319-6A21* | family | 0 | 0.0384 | 0 | |
| *Xanthomonadaceae* | family | 0 | 0.0348 | 1.2347 | |
| *Clostridiaceae* | family | 0 | 0.0295 | 0 | |
| *Ellin6075* | family | 0 | 0.0264 | 0 | |
| *Phyllobacteriaceae* | family | 0 | 0.0247 | 0 | |
| *Chitinophagaceae* | family | 0 | 0 | 8.4041 | |
| *Acidobacteriaceae* | family | 0 | 0 | 0.6705 | |
| *Nitrosomonadaceae* | family | 0 | 0 | 0.1973 | |
| *Actinospicaceae* | family | 0 | 0 | 0.1884 | |
| *Thermomonosporaceae* | family | 0 | 0 | 0.077 | |
| *Syntrophobacteraceae* | family | 0 | 0 | 0.0613 | |
| *Sphingobacteriaceae* | family | 0 | 0 | 0.0529 | |
| *Micromonosporaceae* | family | 0 | 0 | 0.052 | |
| *Patulibacteraceae* | family | 0 | 0 | 0.0514 | |
| *Aerococcaceae* | family | 0 | 0 | 0.0389 | |
| *Burkholderiaceae* | family | 0 | 0 | 0.0386 | |
| *Sinobacteraceae* | family | 0 | 0 | 0.0356 | |
| *AKIW874* | family | 0 | 0 | 0.0336 | |
| *Sporichthyaceae* | family | 0 | 0 | 0.0205 | |
| *Streptophyta* | order | 2.8608 | 8.8638 | 2.5269 | |
| *Bacillales* | order | 0.6657 | 0.0273 | 0 | |
| *CCU21* | order | 0.1972 | 0 | 0 | |
| *Rhizobiales* | order | 0.1708 | 0 | 0.0773 | |
| *Actinomycetales* | order | 0.1145 | 0 | 0.0795 | |
| *Acidimicrobiales* | order | 0.0879 | 0 | 0 | |
| *iii1-15* | order | 0.0843 | 0 | 0 | |
| *RB41* | order | 0.0579 | 0 | 0.0977 | |
| *Ellin329* | order | 0 | 0.2261 | 0.024 | |
| *iii1-15* | order | 0 | 0.1509 | 0 | |
| *WD2101* | order | 0 | 0.0764 | 0.0567 | |
| *JG30-KF-CM45* | order | 0 | 0.0717 | 0 | |
| *Stramenopiles* | order | 0 | 0.0378 | 0 | |
| *Solirubrobacterales* | order | 0 | 0 | 0.3707 | |
| *Phycisphaerales* | order | 0 | 0 | 0.0731 | |
| *Bacteroidales* | order | 0 | 0 | 0.0079 | |
| *Betaproteobacteria* | class | 0.1468 | 0 | 0 | |
| *Acidobacteria-5* | class | 0.0385 | 0 | 0.0441 | |
| *Ellin6529* | class | 0 | 0.1025 | 0.1496 | |
| *Gemm-1* | class | 0 | 0.018 | 0 | |
| *Gemmatimonadetes* | class | 0 | 0 | 0.1005 | |
| *Unclassified* | Unclassified | 11.086 | 1.957 | 4.1025 | |
| *ANCOVA univariate test (P<0.05) | | |  |  | |

| **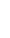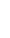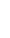Supplementary table S3:** Table format of venn diagrams (A: All treatments, B: *A. thaliana* fed, C: *S.* *lycopersicum* fed, D: Artificial diet treatment), depicting common and unique bacterial ASVs by groups. Groups are denoted in the first column ‘Groups’ and separated by each color for each section of the table. | | |
| --- | --- | --- |
| Venn Diagram A | | |
| Groups | # | Names |
| Common between Populations fed with *A. thaliana* leaves | 34 | Streptococcus |
| and Population fed with *S. lycopersicum* leaves and Population fed with Artificial Diet |  | Microbacteriaceae |
|  |  | Moraxellaceae |
|  |  | Eikenella |
|  |  | Nocardioidaceae |
|  |  | Pseudomonadaceae |
|  |  | Pseudomonas |
|  |  | Corynebacterium |
|  |  | Bradyrhizobiaceae |
|  |  | Mycobacterium |
|  |  | Staphylococcus |
|  |  | Acinetobacter |
|  |  | Erythrobacteraceae |
|  |  | Microbacterium |
|  |  | Comamonadaceae |
|  |  | Enterobacteriaceae |
|  |  | Agrobacterium |
|  |  | Neisseriaceae |
|  |  | Gaiellaceae |
|  |  | Haemophilus |
|  |  | Bacillus |
|  |  | Actinobaculum |
|  |  | Streptophyta |
|  |  | Sphingomonas |
|  |  | Streptomyces |
|  |  | Micrococcaceae |
|  |  | Deinococcus |
|  |  | unclassified |
|  |  | Nocardioides |
|  |  | Propionibacterium |
|  |  | Rhodococcus |
|  |  | Paracoccus |
|  |  | Rhodospirillaceae |
|  |  | Oxalobacteraceae |
| Common between Populations fed with *A. thaliana* leaves and | 24 | Bacillaceae |
| Population fed *S. lycopersicum* leaves |  | mitochondria |
|  |  | Friedmanniella |
|  |  | Moryella |
|  |  | iii1-15 |
|  |  | Bacillales |
|  |  | Rhodoplanes |
|  |  | Enhydrobacter |
|  |  | Mesorhizobium |
|  |  | Intrasporangiaceae |
|  |  | C111 |
|  |  | Pseudonocardia |
|  |  | Arthrobacter |
|  |  | Methylobacterium |
|  |  | Hymenobacter |
|  |  | Sphingomonadaceae |
|  |  | Serratia |
|  |  | Shinella |
|  |  | Xenococcaceae |
|  |  | Weeksellaceae |
|  |  | Rhizobiaceae |
|  |  | Devosia |
|  |  | Cellulomonas |
|  |  | Lactobacillus |
| Common between Population fed with Artificial Diet and | 6 | Rhizobiales |
| Population fed with *A. thaliana* leaves |  | Planococcaceae |
|  |  | Acidobacteria-5 |
|  |  | Enterococcus |
|  |  | RB41 |
|  |  | Lactococcus |
| Common between Population fed with Artificial Diet and | 19 | Solirubrobacterales |
| Population fed with *S. lycopersicum* leaves |  | Facklamia |
|  |  | Ellin329 |
|  |  | WD2101 |
|  |  | Kaistobacter |
|  |  | Streptomycetaceae |
|  |  | Clavibacter |
|  |  | Aerococcaceae |
|  |  | Flavobacterium |
|  |  | Veillonella |
|  |  | Rothia |
|  |  | Gemella |
|  |  | Caulobacteraceae |
|  |  | Granulicatella |
|  |  | Prevotella |
|  |  | Geodermatophilaceae |
|  |  | Rhodanobacter |
|  |  | Xanthomonadaceae |
|  |  | Ellin6529 |
| Only Population fed with A. thaliana | 28 | Ochrobactrum |
|  |  | Acidimicrobiales |
|  |  | Betaproteobacteria |
|  |  | Roseococcus |
|  |  | Sphingobium |
|  |  | Sporomusa |
|  |  | Kaistia |
|  |  | Cryocola |
|  |  | Frankiaceae |
|  |  | Stenotrophomonas |
|  |  | RFP12 |
|  |  | Skermanella |
|  |  | Alcaligenaceae |
|  |  | Novosphingobium |
|  |  | Janibacter |
|  |  | Paenibacillus |
|  |  | Tepidimonas |
|  |  | Glycomyces |
|  |  | Actinomycetales |
|  |  | Variovorax |
|  |  | Kocuria |
|  |  | Promicromonosporaceae |
|  |  | Pirellulaceae |
|  |  | Hyphomicrobiaceae |
|  |  | Terribacillus |
|  |  | Conexibacteraceae |
|  |  | CCU21 |
|  |  | Hyphomicrobium |
| Only Population fed with *S. lycopersicum* leaves | 52 | Brevundimonas |
|  |  | Roseomonas |
|  |  | Solibacillus |
|  |  | Dermacoccus |
|  |  | Denitrobacter |
|  |  | Sphingopyxis |
|  |  | Methylopila |
|  |  | Burkholderia |
|  |  | JG30-KF-CM45 |
|  |  | Dermabacter |
|  |  | Rubrivivax |
|  |  | Delftia |
|  |  | Actinocatenispora |
|  |  | Finegoldia |
|  |  | Aggregatibacter |
|  |  | Streptococcaceae |
|  |  | Leuconostoc |
|  |  | Lautropia |
|  |  | Amaricoccus |
|  |  | Labrys |
|  |  | Methylobacteriaceae |
|  |  | Exiguobacterium |
|  |  | Brevibacterium |
|  |  | Stramenopiles |
|  |  | Thermomonas |
|  |  | Steroidobacter |
|  |  | Amycolatopsis |
|  |  | Luteimonas |
|  |  | Dokdonella |
|  |  | Acetobacter |
|  |  | Anaerococcus |
|  |  | Chryseobacterium |
|  |  | Candidatus_Solibacter |
|  |  | Mycoplana |
|  |  | Clostridiaceae |
|  |  | Actinomyces |
|  |  | 0319-6A21 |
|  |  | Phyllobacteriaceae |
|  |  | Jeotgalicoccus |
|  |  | Acetobacteraceae |
|  |  | Ellin6075 |
|  |  | Peptoniphilus |
|  |  | Halomonadaceae |
|  |  | Paludibacter |
|  |  | Tetrathiobacter |
|  |  | Rhizobium |
|  |  | Gemm-1 |
|  |  | Shewanella |
|  |  | Cellulosimicrobium |
|  |  | Asticcacaulis |
|  |  | Alicyclobacillaceae |
|  |  | Porphyromonas |
| Only Population fed with Artificial Diet | 38 | Phycisphaerales |
|  |  | Oribacterium |
|  |  | Blastococcus |
|  |  | Geobacillus |
|  |  | Couchioplanes |
|  |  | Erwinia |
|  |  | AKIW874 |
|  |  | Rathayibacter |
|  |  | Lysobacter |
|  |  | Luteolibacter |
|  |  | Limnohabitans |
|  |  | Syntrophobacteraceae |
|  |  | Chitinophagaceae |
|  |  | Sinobacteraceae |
|  |  | Actinomadura |
|  |  | Dietzia |
|  |  | Balneimonas |
|  |  | Gluconobacter |
|  |  | Micromonosporaceae |
|  |  | Ramlibacter |
|  |  | Aeromicrobium |
|  |  | Sporichthyaceae |
|  |  | Actinospicaceae |
|  |  | Burkholderiaceae |
|  |  | Cellvibrio |
|  |  | Dyella |
|  |  | Acidobacteriaceae |
|  |  | Nitrosomonadaceae |
|  |  | Thermomonosporaceae |
|  |  | Gemmatimonadetes |
|  |  | Flavisolibacter |
|  |  | Solirubrobacteraceae |
|  |  | Achromobacter |
|  |  | Salinicoccus |
|  |  | Agrococcus |
|  |  | Patulibacteraceae |
|  |  | Bacteroidales |
|  |  | Sphingobacteriaceae |
|  |  |  |
|  |  |  |
|  |  |  |
| Venn Diagram B | | |
| Groups | # | Names |
| Common between *A. thaliana* leaves and | 19 | Streptophyta |
| Population fed with *A. thaliana* leaves |  | Rhizobiales |
|  |  | Moraxellaceae |
|  |  | Streptomyces |
|  |  | Nocardioidaceae |
|  |  | Pseudomonas |
|  |  | mitochondria |
|  |  | Bradyrhizobiaceae |
|  |  | Acinetobacter |
|  |  | Rhodoplanes |
|  |  | Mesorhizobium |
|  |  | unclassified |
|  |  | Propionibacterium |
|  |  | Enterobacteriaceae |
|  |  | Rhodospirillaceae |
|  |  | Hyphomicrobiaceae |
|  |  | Shinella |
|  |  | Rhizobiaceae |
|  |  | Oxalobacteraceae |
| Only *A. thaliana* leaves | 14 | Rhodospirillales |
|  |  | Flavobacterium |
|  |  | Ellin329 |
|  |  | Sphingopyxis |
|  |  | Cellvibrio |
|  |  | Phyllobacteriaceae |
|  |  | Nitrosomonadaceae |
|  |  | Methylibium |
|  |  | OM27 |
|  |  | MND1 |
|  |  | SC-I-84 |
|  |  | Xanthomonadaceae |
|  |  | Methylophilaceae |
|  |  | Dokdonella |
| Only Population fed only with *A. thaliana* leaves | 73 | Ochrobactrum |
|  |  | Streptococcus |
|  |  | Arthrobacter |
|  |  | Acidimicrobiales |
|  |  | Actinobaculum |
|  |  | Novosphingobium |
|  |  | Betaproteobacteria |
|  |  | Janibacter |
|  |  | Sphingomonas |
|  |  | Roseococcus |
|  |  | Microbacteriaceae |
|  |  | Tepidimonas |
|  |  | Paenibacillus |
|  |  | Planococcaceae |
|  |  | Eikenella |
|  |  | Pseudomonadaceae |
|  |  | Methylobacterium |
|  |  | Bacillaceae |
|  |  | Micrococcaceae |
|  |  | Hymenobacter |
|  |  | Friedmanniella |
|  |  | Moryella |
|  |  | Sphingomonadaceae |
|  |  | Glycomyces |
|  |  | Corynebacterium |
|  |  | iii1-15 |
|  |  | Mycobacterium |
|  |  | Staphylococcus |
|  |  | Actinomycetales |
|  |  | Acidobacteria-5 |
|  |  | Erythrobacteraceae |
|  |  | Bacillales |
|  |  | Enterococcus |
|  |  | Deinococcus |
|  |  | Enhydrobacter |
|  |  | Serratia |
|  |  | Microbacterium |
|  |  | Sphingobium |
|  |  | Nocardioides |
|  |  | Variovorax |
|  |  | Kocuria |
|  |  | RB41 |
|  |  | Promicromonosporaceae |
|  |  | Rhodococcus |
|  |  | Pirellulaceae |
|  |  | Comamonadaceae |
|  |  | Sporomusa |
|  |  | Kaistia |
|  |  | Paracoccus |
|  |  | Agrobacterium |
|  |  | Cryocola |
|  |  | Xenococcaceae |
|  |  | Neisseriaceae |
|  |  | Lactococcus |
|  |  | Weeksellaceae |
|  |  | Terribacillus |
|  |  | Frankiaceae |
|  |  | Conexibacteraceae |
|  |  | Intrasporangiaceae |
|  |  | Stenotrophomonas |
|  |  | RFP12 |
|  |  | Gaiellaceae |
|  |  | Devosia |
|  |  | Skermanella |
|  |  | Haemophilus |
|  |  | Alcaligenaceae |
|  |  | Cellulomonas |
|  |  | CCU21 |
|  |  | C111 |
|  |  | Bacillus |
|  |  | Pseudonocardia |
|  |  | Hyphomicrobium |
|  |  | Lactobacillus |
|  |  |  |
|  |  |  |
|  |  |  |
| Venn Diagram C | | |
| Groups | # | Names |
| Common between *S. lycopersicum* leaves and | 14 | Moraxellaceae |
| Population fed with *S. lycopersicum* |  | Pseudomonas |
|  |  | mitochondria |
|  |  | Delftia |
|  |  | Comamonadaceae |
|  |  | Enterobacteriaceae |
|  |  | Agrobacterium |
|  |  | Streptophyta |
|  |  | Chryseobacterium |
|  |  | Sphingomonas |
|  |  | Caulobacteraceae |
|  |  | unclassified |
|  |  | Rhizobium |
|  |  | Xanthomonadaceae |
| Only *S. lycopersicum* leaves | 5 | Sphingobacterium |
|  |  | Methylotenera |
|  |  | Sphingobium |
|  |  | Limnohabitans |
|  |  | Azospirillum |
| Only Population fed with *S. lycopersicum* | 115 | Streptococcus |
|  |  | Solirubrobacterales |
|  |  | Facklamia |
|  |  | Brevundimonas |
|  |  | Microbacteriaceae |
|  |  | Roseomonas |
|  |  | Solibacillus |
|  |  | Dermacoccus |
|  |  | Denitrobacter |
|  |  | Eikenella |
|  |  | Nocardioidaceae |
|  |  | Pseudomonadaceae |
|  |  | Bacillaceae |
|  |  | Ellin329 |
|  |  | Sphingopyxis |
|  |  | Friedmanniella |
|  |  | Methylopila |
|  |  | Moryella |
|  |  | Kaistobacter |
|  |  | WD2101 |
|  |  | Corynebacterium |
|  |  | iii1-15 |
|  |  | Mycobacterium |
|  |  | Bradyrhizobiaceae |
|  |  | Staphylococcus |
|  |  | Acinetobacter |
|  |  | Burkholderia |
|  |  | Erythrobacteraceae |
|  |  | Rhodoplanes |
|  |  | Bacillales |
|  |  | JG30-KF-CM45 |
|  |  | Dermabacter |
|  |  | Enhydrobacter |
|  |  | Streptomycetaceae |
|  |  | Mesorhizobium |
|  |  | Rubrivivax |
|  |  | Microbacterium |
|  |  | Actinocatenispora |
|  |  | Aggregatibacter |
|  |  | Finegoldia |
|  |  | Streptococcaceae |
|  |  | Leuconostoc |
|  |  | Clavibacter |
|  |  | Lautropia |
|  |  | Amaricoccus |
|  |  | Labrys |
|  |  | Methylobacteriaceae |
|  |  | Neisseriaceae |
|  |  | Exiguobacterium |
|  |  | Intrasporangiaceae |
|  |  | Brevibacterium |
|  |  | Gaiellaceae |
|  |  | Haemophilus |
|  |  | Stramenopiles |
|  |  | Thermomonas |
|  |  | Steroidobacter |
|  |  | Bacillus |
|  |  | C111 |
|  |  | Amycolatopsis |
|  |  | Pseudonocardia |
|  |  | Luteimonas |
|  |  | Dokdonella |
|  |  | Aerococcaceae |
|  |  | Acetobacter |
|  |  | Anaerococcus |
|  |  | Arthrobacter |
|  |  | Flavobacterium |
|  |  | Actinobaculum |
|  |  | Candidatus_Solibacter |
|  |  | Veillonella |
|  |  | Rothia |
|  |  | Mycoplana |
|  |  | Streptomyces |
|  |  | Clostridiaceae |
|  |  | Gemella |
|  |  | Methylobacterium |
|  |  | Actinomyces |
|  |  | 0319-6A21 |
|  |  | Micrococcaceae |
|  |  | Hymenobacter |
|  |  | Phyllobacteriaceae |
|  |  | Jeotgalicoccus |
|  |  | Acetobacteraceae |
|  |  | Sphingomonadaceae |
|  |  | Granulicatella |
|  |  | Ellin6075 |
|  |  | Deinococcus |
|  |  | Serratia |
|  |  | Peptoniphilus |
|  |  | Halomonadaceae |
|  |  | Paludibacter |
|  |  | Tetrathiobacter |
|  |  | Nocardioides |
|  |  | Prevotella |
|  |  | Propionibacterium |
|  |  | Rhodococcus |
|  |  | Gemm-1 |
|  |  | Shewanella |
|  |  | Cellulosimicrobium |
|  |  | Paracoccus |
|  |  | Geodermatophilaceae |
|  |  | Rhodospirillaceae |
|  |  | Shinella |
|  |  | Rhodanobacter |
|  |  | Asticcacaulis |
|  |  | Xenococcaceae |
|  |  | Weeksellaceae |
|  |  | Rhizobiaceae |
|  |  | Alicyclobacillaceae |
|  |  | Devosia |
|  |  | Porphyromonas |
|  |  | Cellulomonas |
|  |  | Oxalobacteraceae |
|  |  | Lactobacillus |
|  |  | Ellin6529 |
|  |  |  |
|  |  |  |
|  |  |  |
|  |  |  |
| Venn Diagram D | | |
| Groups | # | Names |
| Common between Artificial Diet and | 21 | Streptophyta |
| Population fed with Artificial Diet |  | Actinobaculum |
|  |  | Aeromicrobium |
|  |  | Moraxellaceae |
|  |  | Nocardioidaceae |
|  |  | Pseudomonas |
|  |  | Geobacillus |
|  |  | Corynebacterium |
|  |  | Acinetobacter |
|  |  | Staphylococcus |
|  |  | Streptomycetaceae |
|  |  | unclassified |
|  |  | AKIW874 |
|  |  | Propionibacterium |
|  |  | Gemmatimonadetes |
|  |  | Enterobacteriaceae |
|  |  | Rhodanobacter |
|  |  | Xanthomonadaceae |
|  |  | Actinomadura |
|  |  | Gaiellaceae |
|  |  | Bacillus |
| Only Artificial Diet | 23 | Lentzea |
|  |  | Sphingomonadales |
|  |  | Peptostreptococcaceae |
|  |  | Chryseobacterium |
|  |  | Paenibacillus |
|  |  | HTCC |
|  |  | mitochondria |
|  |  | Sphingobacterium |
|  |  | Brachybacterium |
|  |  | Rhodoplanes |
|  |  | JG30-KF-CM45 |
|  |  | Serratia |
|  |  | Mesorhizobium |
|  |  | Brevibacillus |
|  |  | Rhodocyclaceae |
|  |  | Ellin6067 |
|  |  | Clostridium |
|  |  | Beijerinckiaceae |
|  |  | Schlegelella |
|  |  | Planctomyces |
|  |  | Exiguobacterium |
|  |  | Plesiocystis |
|  |  | AK1AB1_02E |
| Only Population fed with Artificial Diet | 76 | Micromonosporaceae |
|  |  | Streptococcus |
|  |  | Solirubrobacterales |
|  |  | Flavobacterium |
|  |  | Ramlibacter |
|  |  | Phycisphaerales |
|  |  | Oribacterium |
|  |  | Rhizobiales |
|  |  | Facklamia |
|  |  | Sphingomonas |
|  |  | Veillonella |
|  |  | Sporichthyaceae |
|  |  | Rothia |
|  |  | Microbacteriaceae |
|  |  | Planococcaceae |
|  |  | Actinospicaceae |
|  |  | Streptomyces |
|  |  | Eikenella |
|  |  | Pseudomonadaceae |
|  |  | Gemella |
|  |  | Ellin329 |
|  |  | Caulobacteraceae |
|  |  | Burkholderiaceae |
|  |  | Micrococcaceae |
|  |  | Cellvibrio |
|  |  | Dyella |
|  |  | Blastococcus |
|  |  | WD2101 |
|  |  | Kaistobacter |
|  |  | Granulicatella |
|  |  | Couchioplanes |
|  |  | Acidobacteriaceae |
|  |  | Erwinia |
|  |  | Bradyrhizobiaceae |
|  |  | Mycobacterium |
|  |  | Acidobacteria-5 |
|  |  | Erythrobacteraceae |
|  |  | Enterococcus |
|  |  | Deinococcus |
|  |  | Nitrosomonadaceae |
|  |  | Microbacterium |
|  |  | Rathayibacter |
|  |  | Lysobacter |
|  |  | Luteolibacter |
|  |  | Nocardioides |
|  |  | Limnohabitans |
|  |  | Prevotella |
|  |  | RB41 |
|  |  | Syntrophobacteraceae |
|  |  | Rhodococcus |
|  |  | Thermomonosporaceae |
|  |  | Clavibacter |
|  |  | Comamonadaceae |
|  |  | Paracoccus |
|  |  | Flavisolibacter |
|  |  | Geodermatophilaceae |
|  |  | Agrobacterium |
|  |  | Rhodospirillaceae |
|  |  | Solirubrobacteraceae |
|  |  | Neisseriaceae |
|  |  | Chitinophagaceae |
|  |  | Lactococcus |
|  |  | Achromobacter |
|  |  | Salinicoccus |
|  |  | Agrococcus |
|  |  | Sinobacteraceae |
|  |  | Dietzia |
|  |  | Balneimonas |
|  |  | Patulibacteraceae |
|  |  | Haemophilus |
|  |  | Gluconobacter |
|  |  | Bacteroidales |
|  |  | Oxalobacteraceae |
|  |  | Sphingobacteriaceae |
|  |  |  |
|  |  |  |
|  |  |  |

**Supplemental Table 4:** KEGG orthologues selected for PICRUSt analysis of predicted bacterial gene function.

| Process | KEGG | | |
| --- | --- | --- | --- |
|  | Gene | Entry | Definition |
| Glycosidases; i.e. enzymes that hydrolyze O- and S-glycosyl compounds | E3.2.1.21 | K01176 | alpha-amylase |
|  |  | K07405 |  |
|  |  | K05343 |  |
|  | E3.2.1.24 | K01191 | alpha-mannosidase |
|  |  | K12311 |  |
|  |  | K12312 |  |
|  | E3.2.1.31 | K14756 | beta-glucuronidase |
|  |  | K01195 |  |
|  | E3.2.1.40 | K05989 | alpha-L-rhamnosidase |
|  | EC.3.2.1.41 | K01200 | pullulanase |
|  | E3.2.1.50 | K01205 | alpha-N-acetylgulcosaminidase |
|  | E3.5.1.28 3.2.1.96 | K13714 | Bifunctional autolysin |
|  | E3.2.1.96 | K01227 | Mannosyl-glycoprotein endo-beta-N-acetylglucosaminidase |
